# On Shell Closure and Beyond: Classification and Functional Interpretation of Chela Types in Paguroidea (Decapoda)

**DOI:** 10.1002/jmor.70161

**Published:** 2026-08-18

**Authors:** Yannic C. Ege, Christian Foth, Stefan Richter

**Affiliations:** ^1^ Allgemeine & Spezielle Zoologie, Institut für Biowissenschaften Universität Rostock Rostock Germany; ^2^ Museum für Naturkunde, Leibniz‐Institut für Evolutions—und Biodiversitätsforschung Berlin Germany; ^3^ Institute for Geological Sciences Freie Universität Berlin Berlin Germany

**Keywords:** 3D imaging, chela shape, evolutionary adaptation, functional morphology, geometric morphometrics, hermit crabs, Paguroidea, shell closure

## Abstract

In Paguroidea, the chelae of the first thoracopods (chelipeds) encompass heterochelate (unequal), homoiochelate (subequal, weakly differentiated), and homochelate (equal, mirrored) conditions, reflecting functional differentiation and evolutionary adaptation across taxa. For 30 species from seven hermit crab families detailed 3D models of chelae were generated using microCT imaging. For these, 3D shape analysis identified six distinct shape types (I—compact, II— sturdy, III—elongate, IV—scutiform, V—discoid, VI—semidiscoid) across the investigated taxa. These shape types correspond closely with key biological roles. Paired *semidiscoid* chelae enable symmetrical shelter closure, while *scutiform* and *discoid* chelae function in one‐sided shelter closure and defense and are associated with the utilization of gastropod shells. *Compact* and *elongate* chelae serve in food manipulation and grooming. *Sturdy* chelae, which include most homoiochelate forms, are associated with intermediate conditions related to alternative defensive strategies, such as complete (deep) withdrawal into shells, preference for shells with narrow apertures, or symbiotic relationships with anthozoans. Phylogenetic patterns might indicate that potentially basal taxa like Pylochelidae retain plesiomorphic, symmetrical semidiscoid chelae, but it cannot be excluded that these homochelate chelae represent an apomorphic condition for the taxon. In any case, the asymmetric hermit crabs (Parapaguridae, Paguridae, Diogenidae, Coenobitidae, Lithodidae) evolved pronounced heterochely, with the dominant chela adapted (primarily) for shelter closure and the subdominant one for feeding and grooming. Homoiochelate forms occur repeatedly across Diogenidae, suggesting multiple evolutionary transitions related to shifts in defensive strategy. The association between chela shape, biological role, and phylogeny highlights the dynamic interplay of evolutionary history, functional demands, and ecological adaptation in shaping hermit crab morphology.

## Graphical abstract

MicroCT technology and 3D shape analysis were used to classify paguroid chelae into six distinct shape types: *compact*, *sturdy*, *elongate*, *scutiform*, *discoid*, and *compact*. Each type is linked to characteristic biological roles: paired semidiscoid chelae enable symmetrical shelter closure; scutiform and discoid chelae support one‐sided shelter closure; compact and elongate chelae associate with food manipulation and grooming; sturdy chelae correspond to alternative defensive strategies (e.g., narrow‐aperture shells, anthozoan symbioses). Phylogenetic patterns indicate multiple independent transitions between symmetric and asymmetric forms across lineages, with clades showing diverse states including retained homochely and the derived conditions heterochely and homoiochely. These results highlight how form, function, and phylogeny interact to shape hermit crab chela diversity.

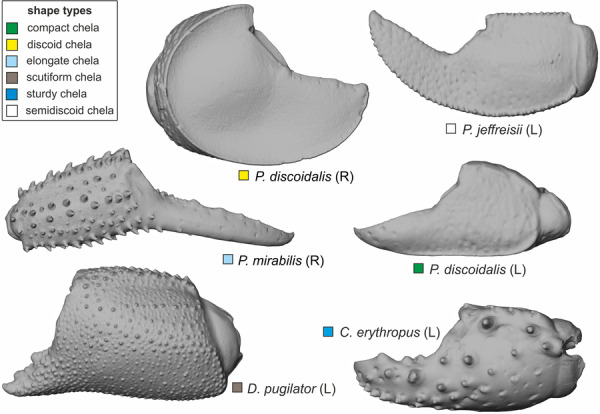

## 1 Introduction

### 1.1 Chelae as Multifunctional Organs in Decapods

In decapod crustaceans, the chelae of the first thoracopods, that is, the chelipeds are involved in various biological tasks (biological roles *sensu* Bock and von Wahlert 1965) such as feeding, defense, agonistic interactions, grooming, and reproduction (Schäfer 1954; Hazlett 1974; Schembri 1982; Mariappan et al. 2000; Laidre and Elwood 2008; Edmonds and Briffa 2016). Chelae disparity qualifies them as models for the study of morphological adaptation and evolutionary constraints. Within this framework, chelae of hermit crabs are of particular interest because they combine the general roles of decapod chelae with unique requirements imposed by the use of external shelters, mostly gastropod shells, for protection (Imafuku 1983; Gruner et al. 1993; Poore and Ahyong 2023).

Hermit crabs *s.l*. (Paguroidea), that is, also including king and stone crabs, occur from shallow marine waters to the deep sea, in terrestrial habitats, in estuaries, and even freshwater (Gruner et al. 1993; McLaughlin and Murray 1990; Osawa and Fujita 2008; Poore and Ahyong 2023), reflecting remarkable ecological diversity. The large quantity of various habitats is matched by a considerable disparity in the chelae, making the group an important model for investigating how form–function complexes evolve under interacting functional and phylogenetic conditions.

The *cutting edges* of chelae, defined by the dentition and arrangement of teeth on the movable and immovable fingers, are closely linked to specific biological roles such as food manipulation and consumption (Schäfer 1954), whereas *overall chela shape* is associated with biological roles beyond feeding. Dominant (larger) chelae frequently fulfill additional roles such as shelter closure, mate attraction, and are used in territorial fights (Reese 1962; Laidre 2012a). Distinguishing the biological roles of the *chela cutting edges* on the one hand and *general chela shape* on the other hand is therefore crucial for understanding the evolutionary diversification of chelae within Paguroidea.

### 1.2 Phylogenetic Relationships of Paguroidea

With more than 1400 described species, Paguroidea represents the most species‐rich taxon within Anomura, comprising over 40% of species currently recognized (De Grave et al. 2009; Lemaitre and McLaughlin 2009; McLaughlin et al. 2010; Almón et al. 2025). Whereas the monophyly of Anomura is widely accepted (Scholtz 1995; Ahyong and O'Meally 2004; Schram et al. 2003; McLaughlin et al. 2007; Reimann et al. 2011), the internal relationships among taxa as well as the monophyly of Paguroidea remain debated (Ahyong et al. 2009; Bracken‐Grissom et al. 2013; Wolfe et al. 2019).

Within Paguroidea, the Pylochelidae are considered representing the ancestral symmetric body form (Richter and Scholtz 1994; McLaughlin and Lemaitre 2009; Reimann et al. 2011). While traditionally regarded as sister group to the remaining hermit crabs, their position has been questioned by phylogenetic analyses. In some studies, both symmetrical Pylochelidae and the deep‐sea‐living Parapaguridae are indicated to be more closely related to squat lobsters (e.g., Galatheidae, Munididae) than to other hermit crab lineages, suggesting that Paguroidea as traditionally defined may be polyphyletic (Ahyong et al. 2009; Tsang et al. 2011; Bracken‐Grissom et al. 2013; Wolfe et al. 2019; Gong et al. 2020).

The remaining paguroid lineages display asymmetry and include the diverse families Diogenidae, Coenobitidae, Paguridae, and Lithodidae. Strong molecular and morphological evidence supports a monophyletic grouping of Paguridae and Lithodidae, with the latter representing king and stone crabs that evolved from right‐handed hermit crab ancestors (Tsang et al. 2011; Bracken‐Grissom et al. 2013; Keiler et al. 2015). Semiterrestrial Coenobitidae are nested within left‐handed Diogenidae (Tsang et al. 2011; Bracken‐Grissom et al. 2013). Diogenidae might even be polyphyletic (Tsang et al. 2011; Bracken‐Grissom et al. 2013; Wolfe et al. 2019), and parts of Paguridae have been reassigned to the newly proposed family Xylopaguridae (Gašparič et al. 2016).

Despite these recent suggestions of polyphyletic Paguroidea, for the sake of the argument and in the line of Poore and Ahyong (2023), we keep the group as a natural unit until we know more about their phylogeny. Also, we retain Diogenidae and Parapaguridae in their traditional sense but recognize Xylopaguridae as a separate family.

### 1.3 Shelter Occupation as Evolutionary Strategy

A key feature of hermit crabs is their occupation of external shelters. This shelter‐based defense strategy allows shelter‐dwelling taxa a reduced investment in pleonal calcification (Przibram 1907).

**Figure 1:**
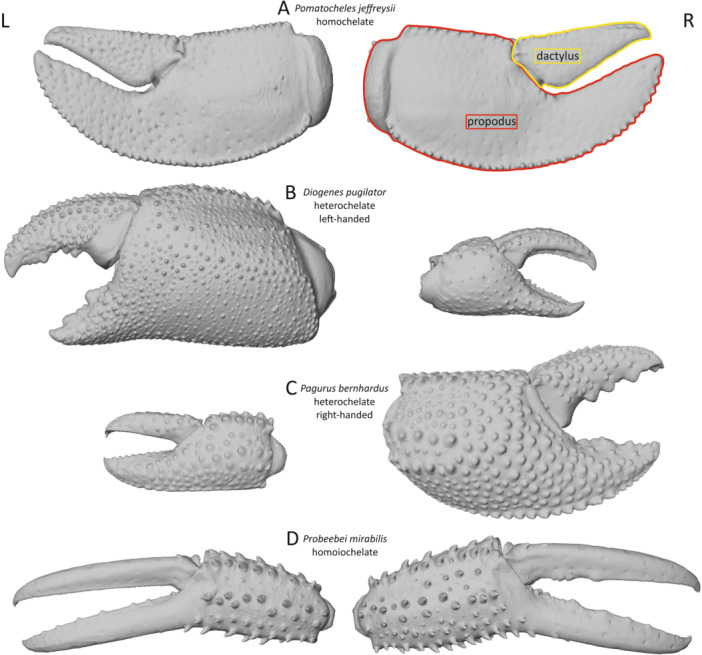
Illustration of the three chela symmetry categories: (A) homochelate chelae of *Pomatocheles jeffreysii* (Pylochelidae) (equal), (B) heterochelate chelae of *Diogenes pugilator* (unequal) (Diogenidae), (C) heterochelate chelae of *Pagurus bernhardus* (unequal) (Paguridae), and (D) homoiochelate chelae of *Probeebei mirabilis* (subequal) (Parapaguridae). Chelae within each pair are correctly scaled relative to one another, but scaling between pairs is not comparable. Podomeres of a chela are framed yellow (dactylus) and red (propodus). L, left chelae; R, right chelae.

In most heterochelate lineages (e.g., Paguridae, Diogenidae, Coenobitidae), which predominantly occupy gastropod shells (Tudge et al. 2012), the dominant chela functions as an operculum for aperture closure (Magnus 1961; Reese 1969; Dowds and Elwood 1983; Elwood and Stewart 1985). This chela is right‐sided in Paguridae and Parapaguridae (Forest 1987a; McLaughlin 2003), but left‐sided in Diogenidae and Coenobitidae (Dowds and Elwood 1983; Elwood and Stewart 1985; Tudge et al. 2012). Therefore, heterochely (Greek: ἕτερος = unequal), that is, clear morphological differentiation of the left and right chela (Przibram 1905), appears to have evolved independently at least twice, in association with an operculiform dominant chela and adaptation to shell‐dwelling (Ege et al. 2025). Some Diogenidae and Parapaguridae instead show homoiochelate (Greek: ὁμοῖος = subequal) chelae, difficult to separate into a dominant and subdominant one, while most Pylochelidae possess homochelate (Greek: ὁμός = equal) chelae, identical in size and shape but mirrored (Figure 1).

Other lineages deviate drastically from classical shell use. Coenobitidae actively modify gastropod shells into long‐lasting, spacious shelters that may persist and are inhabited over multiple generations (Laidre 2012a, 2012b). Lithodidae, the closest relatives (or even ingroup) of Paguridae, represent the reverse transformation, abandoning shells and secondarily acquiring a heavily calcified pleon in association with carcinization, the evolution toward a crab‐like body form (Richter and Scholtz 1994; Scholtz 2014; Keiler et al. 2015).

Homochely, together with a symmetric body form, has been proposed as the ancestral condition of Paguroidea (Richter and Scholtz 1994; McLaughlin and Lemaitre 2009; Reimann et al. 2011), a scenario supported by fossil evidence from the Early and Middle Jurassic, which suggests that homochelate chelae represent the plesiomorphic condition within the group (Schweitzer et al. 2009; Fraaije et al. 2013). Distinct heterochely, in contrast, appears only in the Late Jurassic (Fraaije et al. 2022).

### 1.4 Objectives of This Study

We acquired detailed 3D models of paguroid chelae using microCT imaging, which provides high‐resolution volumetric data. These models were then processed and analyzed using spherical‐harmonics‐based 3D shape analysis (Foth et al. 2019; Ege et al. 2020, 2025), a method that enables comprehensive quantification of complex shapes beyond the limitations of traditional 2D outline analyses or 3D landmark‐based approaches. This approach describes closed 3D surfaces as a weighted sum of spherical harmonic basis functions, comparable to a Fourier decomposition. By applying *SPHARM* (Shen and Makedon 2006; Shen et al. 2009 *)*, we provide a comprehensive 3D data set that allows testing for hypotheses about form–function integration and the influence of phylogeny on the shape of hermit crab chelae.

The objectives were threefold.

1. To classify paguroid chelae into distinct *shape types* with reference to different symmetry categories: homochelate (mirrored, symmetrical chelae), heterochelate (distinct dominant and subdominant chelae), and homoiochelate (not readily distinguishable as dominant vs. subdominant chelae).
2. To relate these chela shape types to key biological roles, primarily shelter closure and food manipulation.
3. To examine how chela shape types and associated biological roles correlate with recent phylogenetic hypotheses within Paguroidea.

## 2 Materials and Methods

### 2.1 Studied Species

We studied the chelae of 33 specimens of 30 paguroid species from seven families:

**Coenobitidae (2)**: *Coenobita rugosus* H. Milne Edwards, 1837; *Coenobita scaevola* (Forskål, 1775). **Diogenidae (9)**: *Cancellus spongicola* Benedict, 1901; *Clibanarius erythropus* (Latreille, 1818); *Diogenes edwardsii* (De Haan, 1849); *Diogenes pugilator* (Roux, 1829); *Isocheles pilosus* (Holmes, 1900); *Paguristes digueti* Bouvier, 1893; *Paguristes eremita* (Linnaeus, 1767); *Paguristes streaensis* Pastore, 1984; *Paguropsis andersoni* (Alcock, 1899). **Lithodidae (4)**: *Cryptolithodes sitchensis* Brandt, 1853; *Dermaturus mandtii* Brandt, 1850; *Hapalogaster grebnitzkii* Schalfeew, 1892; *Hapalogaster mertensii* Brandt, 1850. **Paguridae (8)**: *Agaricochirus erosus* (A. Milne‐Edwards, 1880); *Manucomplanus spinulosus* (Holthuis, 1959); *Pagurus anachoretus* Risso, 1827; *Pagurus beringanus* (Benedict, 1892); *Pagurus bernhardus* (Linnaeus, 1758); *Pagurus cuanensis* Bell, 1845; *Phimochirus holthuisi* (Provenzano, 1961); *Pylopagurus discoidalis* (A. Milne‐Edwards, 1880). **Parapaguridae (3)**: *Parapagurus pilosimanus* Smith, 1879; *Probeebei mirabilis* Boone, 1926; *Sympagurus dimorphus* (Studer, 1883). **Pylochelidae (3)**: *Bathycheles integer* (Forest 1987a); *Pomatocheles jeffreysii* Miers, 1879; *Trizocheles boasi* Forest 1987a. **Xylopaguridae (1)**: *Xylopagurus cancellarius* A. Milne‐Edwards, 1880.

A full list of the studied specimens (which are catalogued and stored either in the National Museum of Natural History, Smithsonian Institution, Washington D.C., USA or in the Zoological Collection of the University of Rostock, Germany) including symmetry categories and handedness is provided in Supporting Information: Table S1.

### 2.2 Specimen Preparation and MicroCT Scanning

In preparation for microCT scanning, chelipeds were dissected from the specimens, and dried to enhance the contrast in CT images and to prevent wobbling artefacts during the scanning process. We omitted further contrasting methods, since the contrast of dried, calcified cuticles is sufficient for shape analyses using microCT (Ege et al. 2020, 2025). X‐ray imaging was conducted using a Phoenix Nanotom‐180 (Phoenix|x‐ray, GE Sensing & Inspection Technologies) high‐resolution MicroCT system or a ZEISS Xradia 410 Versa (Carl Zeiss X‐ray Microscopy Inc., Pleasanton, USA).

### 2.3 Segmentation

3D reconstruction and segmentation of the chelae was performed based on virtual image stacks generated by microCT, using the software *Amira 3D* v.2021.2 (FEI Visualization Sciences Group, Zuse Institute Berlin). Podomeres of the chelae (dactylus and propodus, respectively) were separated. Four left propodi were damaged and therefore excluded from the study (*C. rugosus*, *D. mandtii*, *P. beringanus* and *T. boasi*). The dactyli were not further investigated, as they closely mirror the corresponding immovable finger (pollex) and therefore provide little additional shape information. Hence, as done in various studies on decapod chela shape using geometric morphometrics or spherical harmonics (Silva and Paula 2008; Simanjuntak and Eprilurahman 2019; Nascimento et al. 2024; Ege et al. 2025), the following analyses were conducted based on the reconstructed propodi and subsequently related to the corresponding chelae. The articulation sockets of the propodi were digitally closed since spherical‐harmonics‐based approaches only work with closed triangular mesh surfaces (Shen and Makedon 2006; Shen et al. 2009). To close the surfaces, the application of an ambient‐occlusion‐based (AO) segmentation algorithm was carried out using the *XImagePAQ* extension for *Amira 3D*. For best results, the AO value of the algorithm was set to 0.7. Further 3D mesh processing steps (including mirroring of the right chelae onto the left side) were generally performed as described by Ege et al. (2020) using *MeshLab* v.2023.12 (Cignoni et al. 2008). This mirroring was performed to enable a shape‐dependent comparison between left and right chelae, ensuring that observed differences reflect true biological asymmetry rather than arbitrary orientation. In the following manuscript, chelae will be referenced; however, all analyses and interpretations are based on propodi.

### 2.4 Shape Analysis

The spherical‐harmonics‐based shape analyses (including a principal component analysis) and the determination of centroid sizes of the 3D meshes were conducted using *SPHARM* v.1.4 (Shen and Makedon 2006; Shen et al. 2009), following the workflow described by Ege et al. (2020). The left chela of *H. grebnitzkii* was sorted out, as its mesh exhibited shape artifacts. Shape analyses were therefore carried out using 61 chelae (28 left and 33 right).

Principal component analysis (PCA) provided the data matrix (eigenvalues for each chela) which was the base for further analyses. Those PCs explaining 95% of the total variance were selected as substantial PCs (Jackson 1993) and used in all subsequent multivariate statistical analyses, which were conducted using *PAST* v4.06b (Hammer et al. 2001).

### 2.5 Multivariate Statistical Analyses

In heterochelate taxa dominant and subdominant chelae differ substantially in shape. We therefore treated them as distinct morphological units although belonging to the same individual. In homoiochelate taxa, this shape difference is less pronounced but still present. In homochelate taxa, chelae are indeed mirrored, but were nonetheless treated as distinct units for consistency.

Depending on the specific research question, different multivariate statistical methods were applied to assess shape variation and group structure, including Ward's cluster analysis, MANOVA, PERMANOVA, and linear discriminant analysis (LDA). Ward's hierarchical clustering method identifies groups by minimizing within‐group variance at each step of the clustering process (Ward 1963). It is particularly effective for generating compact and homogeneous clusters (Everitt and Hothorn 2011; Hammer and Harper 2006). Following Ege et al. (2025), for MANOVA the number of dependent variables (constraints) was set to six to optimize *p*‐values, as suggested for Procrustes‐fitted 3D data (Hammer et al. 2001). In contrast to parametric analyses, PERMANOVA does not require normally distributed data (Hammer and Harper 2006). Here, we used 9999 iterations and the Mahalanobis distance, which is more robust to statistical outliers than Euclidean distance, as it also accounts for the shape of the data distribution and enables precise distance measurements for correlated variables (Mahalanobis 1936; Etherington 2021). For both MANOVA and PERMANOVA, the spatial relationship between tested groups was expressed by an *F*‐value and a Bonferroni‐corrected *p*‐value, when more than two groups were compared. *p*‐values of 0.05 and below were considered statistically significant. Throughout multivariate methods, *fail* refers to cases of insufficient sample size for MANOVA, whereas *NAN* (not a number) refers to mathematical artifacts arising from non‐normally distributed data groups in PERMANOVA (Hammer et al. 2001; Zelditch et al. 2012). LDA provided a discriminant analysis for two or more groups, reducing redundant and noisy information (Zhang et al. 2024). When comparing at least three groups, this resulted in plots along the first two canonical axes, achieving maximum and second‐to‐maximum separation among all groups. To assess group similarity, the classifier function of LDA was used to estimate group assignments for each data point (chela, respectively). These assignments were validated through jackknife cross‐validation (Hammer and Harper 2006; Mitteroecker and Bookstein 2011). When necessary, distances between group centroids were calculated using Euclidean distance and the centroid coordinates obtained from LDA.

To classify the chelae into distinct shape types based on morphological similarity, Ward's cluster analysis was applied to the original data set (61 chelae). Identified shape patterns were used to explore whether a distinct shape type corresponds to biological roles or systematic groupings. To further test the significance and robustness of the shape types, MANOVA, PERMANOVA, and LDA were applied.

To investigate whether the shape types correspond to functional or systematic classifications, previously mentioned statistical methods (MANOVA, PERMANOVA and LDA) were applied using specific classification criteria on the chelae. These criteria resulted in the series of the following tests no. 1 to 6. For clarity a table of the tests is provided in Supporting Information: Table S2.

1. First, a side‐specific classification of the chelae at family level was conducted and tested. Due to their homochely, pylochelid chelae were represented as one group. Since a minimum group size was required, only the following eight groups could be compared, resulting in 54 chelae of the original data set being included in the comparison: Diogenidae (dominant = dom), Diogenidae (subdominant = sdom), Lithodidae (dom), Paguridae (dom), Paguridae (sdom), Parapaguridae (dom), Parapaguridae (sdom), and Pylochelidae. The following groups (seven chelae in total) were excluded: Lithodidae (sdom), Xylopaguridae (dom) and (sdom), Coenobitidae (dom) and (sdom).
2. In a next step, we examined whether dominant and subdominant chelae (hetero‐ and homoiochelate) across left‐handed and right‐handed species (therefore without the five pylochelid chelae) are more similar to each other than all right chelae vs. all left chelae (two groups; 56 chelae).
3. Following this, homochelate (semidiscoid) chelae of the Pylochelidae were analyzed to determine whether they occupy a unique or intermediate position within the data set, or whether they align more closely with the subdominant or dominant chelae of the other taxa (three groups; 60 chelae). The chela of *T. boasi* was excluded because it did not fall within the same shape type.
4. Next, a distinct new group was formed with taxa displaying homoiochely (*cf*. Supporting Information: Table S1), in which dominant and subdominant pairs of chelae could not or hardly be separated (12 chelae in total). The aim was to determine whether they constitute a separate group compared to the heterochelate dominant and subdominant chelae, as well as their relationship to taxa displaying homochely (four groups; 60 chelae). The chela of *T. boasi* was excluded.
5. In the following, we examined shape differences within all dominant and all subdominant chelae in relation to their side‐specific occurrence. The starting point was the original taxon sampling excluding homochelate chelae (as in test no. 2). To characterize these differences, systematic exclusion steps were applied.
  A. Shape differences within dominant and subdominant chelae (hetero‐ and homoiochelate taxa) in relation to their side‐specific occurrence (four groups; 56 chelae).
  B. Here, the taxon sampling was adjusted by excluding Lithodidae and Coenobitidae. This way we tested for a systematic signal within hetero‐ and homoiochelate taxa that are mostly shell dwellers but without shell modification (four groups; 47 chelae).
  C. Now, we further excluded Parapaguridae. This way we tested for a systematic signal within hetero‐ and homoiochelate taxa that are solely shell dwellers without shell modification (four groups; 41 chelae).

6. Finally, a more detailed test of the positions of homo‐ and homoiochelate chelae within the data set was done (*cf*. test no. 4). To ensure consistency, the chela of *T. boasi* was excluded, resulting in a homochelate group that exclusively contained semidiscoid chelae. The remaining 60 chelae were categorized into side‐specific dominant and subdominant groups, yielding a total of six distinct groups: homochelate, homoiochelate, dominant left, dominant right, subdominant left and subdominant right.

## 3 Results

### 3.1 Shape Type Classification

**Figure 2:**
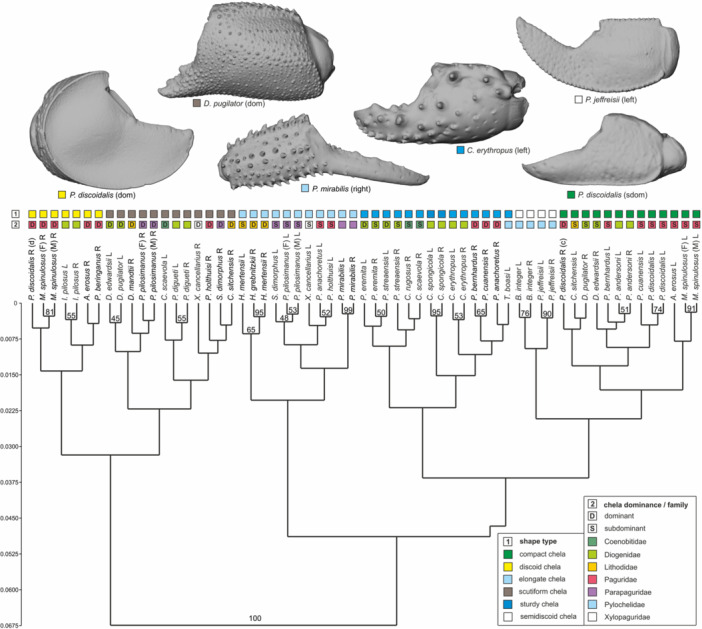
Ward's cluster analysis of the original data set containing 61 chelae. (1) shape type classification; (2) side‐specific classification at family level, empty squares represent homoiochelate or, in the case of *Bathycheles integer* and *Pomatocheles jeffreysii*, homochelate chelae. Examined *Pylopagurus discoidalis* individuals differ in the shape of their dominant (right) chela (c, compact; d, discoid). dom or (D), dominant L, left chela; R, right chela; dominant; sdom or (S), subdominant. *Note:* Size of exemplified chelae (propodi, respectively) is not represented to scale.

**Figure 3:**
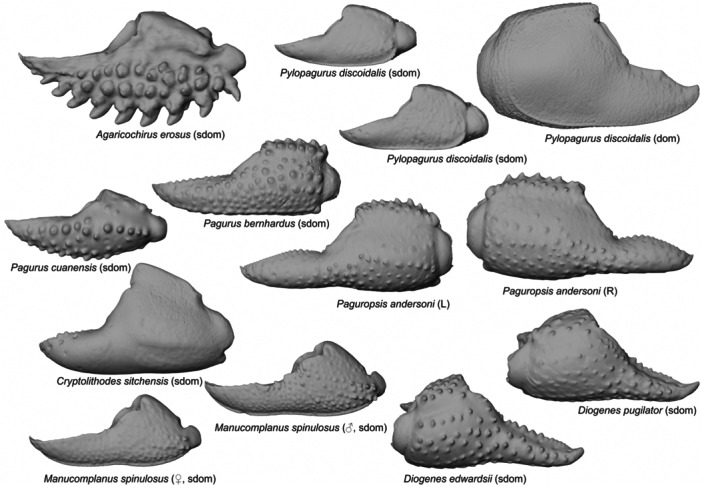
Depiction of propodi assigned to shape type I (compact chela). In heterochelate taxa, chela pairs are labeled as dominant (dom) and subdominant (sdom). In homo‐ and homoiochelate taxa, chelae are distinguished by left (L) and right (R). *Note:* The size of exemplified chelae is not to scale.

**Figure 4:**
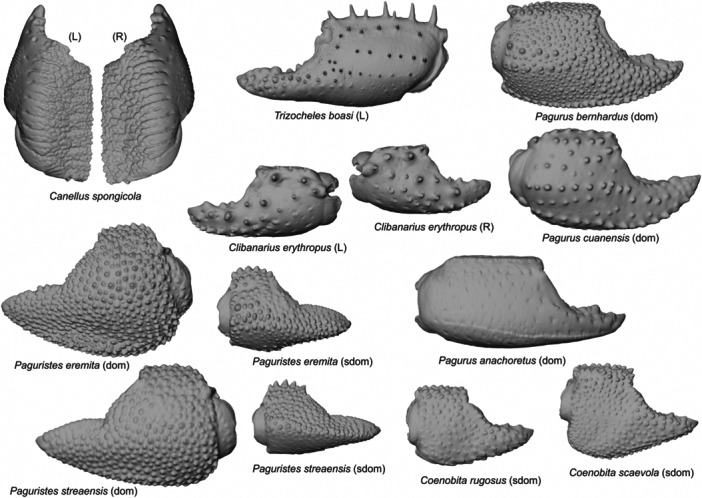
Depiction of propodi assigned to shape type II (sturdy chela). In heterochelate taxa, chela pairs are labeled as dominant (dom) and subdominant (sdom). In homo‐ and homoiochelate taxa, chelae are distinguished by left (L) and right (R). *Note:* The size of exemplified chelae is not to scale.

**Figure 5:**
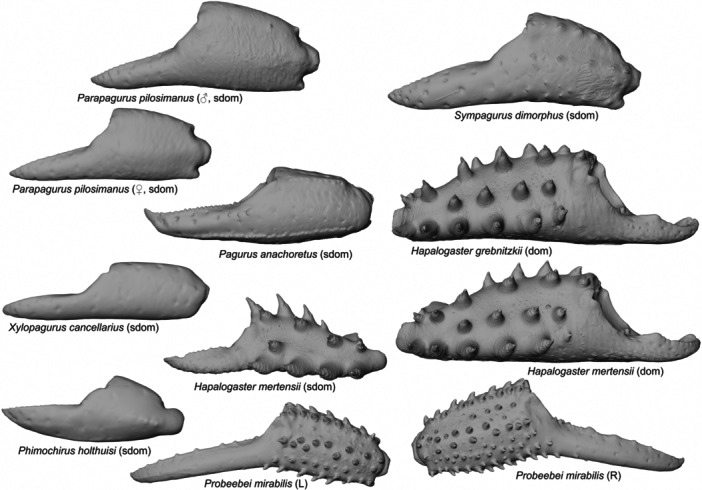
Depiction of propodi assigned to shape type III (elongate chela). In heterochelate taxa, chela pairs are labeled as dominant (dom) and subdominant (sdom). In homo‐ and homoiochelate taxa, chelae are distinguished by left (L) and right (R). The size of exemplified chelae is not to scale.

**Figure 6:**
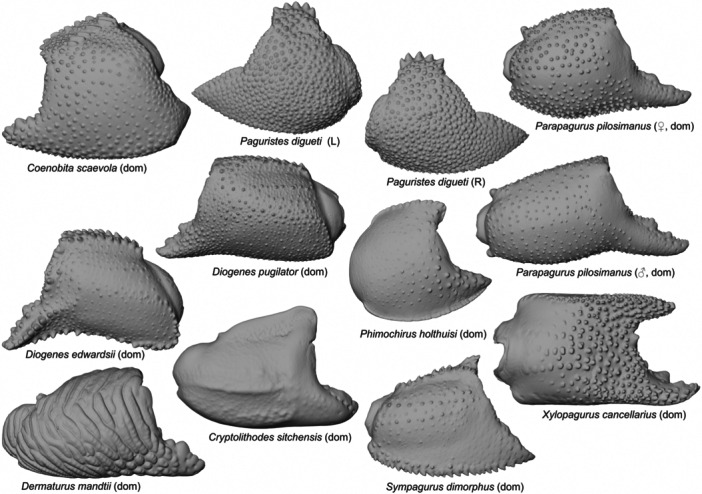
Depiction of propodi assigned to shape type IV (scutiform chela). In heterochelate taxa, chela pairs are labeled as dominant (dom) and subdominant (sdom). In homo‐ and homoiochelate taxa, chelae are distinguished by left (L) and right (R). The size of exemplified chelae is not to scale.

**Figure 7:**
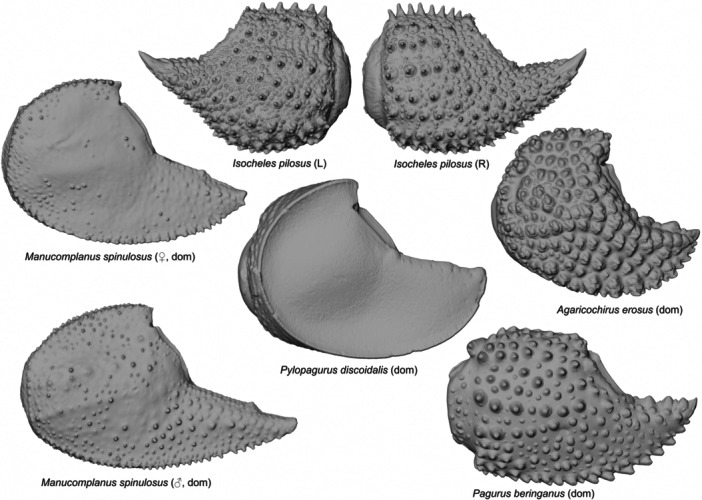
Depiction of propodi assigned to shape type V (discoid chela). In heterochelate taxa, chela pairs are labeled as dominant (dom) and subdominant (sdom). In homo‐ and homoiochelate taxa, chelae are distinguished by left (L) and right (R). The size of exemplified chelae is not to scale.

**Figure 8:**
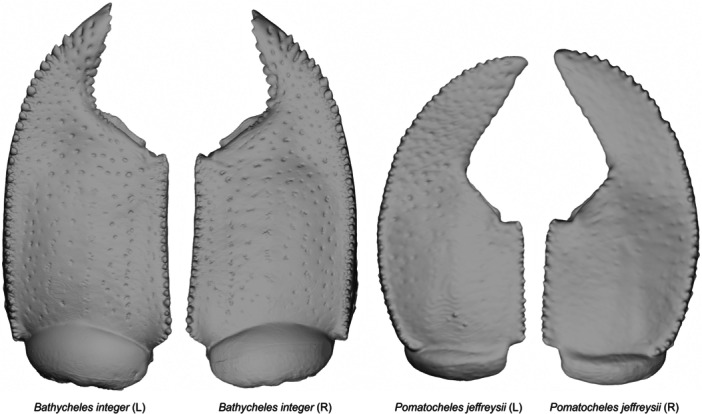
Depiction of propodi assigned to shape type VI (semidiscoid chela). In heterochelate taxa, chela pairs are labeled as dominant (dom) and subdominant (sdom). In homo‐ and homoiochelate taxa, chelae are distinguished by left (L) and right (R). The size of exemplified chelae is not to scale.

**Figure 9:**
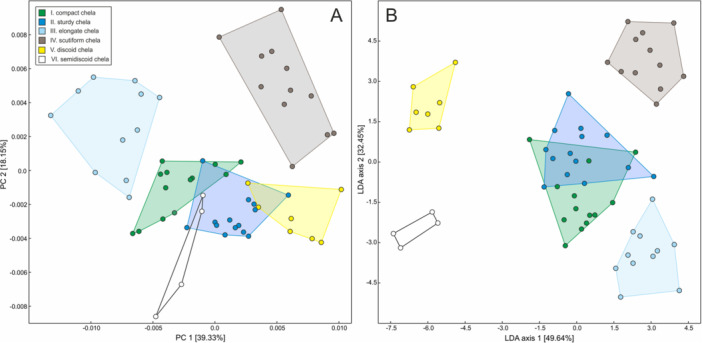
(A) Scatterplot of PC1 and PC2 of the original data set (61 chelae). (B) Scatterplot of LDA axes 1 and 2 of the original data set. Figures display the classification of chelae into the six shape types. According to LDA, 98.4% of the analyzed chelae were correctly classified into shape types, with classification accuracy reduced to 86.9% after jackknifing. Unlike other pylochelid chelae (shape type VI), *T. boasi* was assigned to shape type II.

PCA of the original data set (61 chelae) found PCs 1 to 16 accounting for 95% of total variance (Supporting Information: Table S3); those PCs were used for all subsequent statistical analyses. Ward's cluster analysis identified six distinct shape types (Figure 2), which were delineated by combining the cluster analysis with the overall shape of the assigned chelae (Figures 3, 4, 5, 6, 7, 8) and were subsequently statistically tested (Figure 9, Table 1).

**Table 1:** Results of the MANOVA (A) and one‐way PERMANOVA (B) of the shape types classification (I to VI). *F*‐values are colorized green, *p*‐values orange, and squared Mahalanobis distances (*MD^2^*) blue. Overall *F*‐values can be found in the top‐left cell. *Fail* entries refer to insufficient sample size, while *NAN* (not a number) refers to mathematical artifacts resulting from non‐normally distributed data groups. Significant differences between groups are marked with bold *p*‐values.

	I	II	III	IV	V	VI
**A *F*: 15.18**						
I (*n* = 13)	*p*‐value	**0.024**	**0.030**	**< 0.001**	**0.006**	*fail*
II (*n* = 14)	12.314		**< 0.001**	**< 0.001**	**0.003**	**0.044**
III (*n* = 11)	16.781	33.673		**< 0.001**	**0.005**	*fail*
IV (*n* = 12)	38.602	32.914	54.290		**0.003**	*fail*
V (*n* = 7)	55.370	52.774	107.740	84.670		*fail*
VI (*n* = 4)	*fail*	73.552	*fail*	*fail*	*fail*	*MD^2^*
**B *F*: 2.94**						
I (*n* = 13)	*p*‐value	**0.014**	**0.028**	**0.003**	0.165	1
II (*n* = 14)	1.535		**0.003**	**0.002**	0.558	1
III (*n* = 11)	1.397	1.497		**0.047**	0.411	**0.002**
IV (*n* = 12)	1.488	1.557	1.349		0.431	1
V (*n* = 7)	1.191	1.222	1.067	1.128		**0.002**
VI (*n* = 4)	1	1.066	*NAN*	0.947	*NAN*	*F*‐value

Except for Pylochelidae, which represent the only members of shape type VI (4 out of 5 chelae), no clear correlation between shape types and systematic classification was detected (Figure 2). However, one relationship is evident: shape types IV (scutiform) and V (discoid) predominantly comprise dominant chelae, while shape type I (compact) is mostly formed by subdominant chelae.

Classification of examined chelae to the six shape types:

**I. compact chela (13 chelae; Figure** **3** **):** *Agaricochirus erosus* (L), *Cryptolithodes sitchensis* (L), *Diogenes edwardsii* (R), *Diogenes pugilator* (R), *Manucomplanus spinulosus* (L‐♀; L‐♂), *Paguropsis andersoni* (L; R), *Pagurus bernhardus* (L), *Pagurus cuanensis* (L), *Pylopagurus discoidalis* (L‐c; L‐d), *Pylopagurus discoidalis* (R‐c).

**II. sturdy chela (14 chelae; Figure** **4** **):** *Cancellus spongicola* (L; R), *Clibanarius erythropus* (L; R), *Coenobita rugosus* (R), *Coenobita scaevola* (R), *Pagurus anachoretus* (R), *Pagurus bernhardus* (R), *Pagurus cuanensis* (R), *Paguristes eremita* (L; R), *Paguristes streaensis* (L; R), *Trizocheles boasi* (L).

**III. elongate chela (11 chelae; Figure** **5** **):** *Hapalogaster grebnitzkii* (R), *Hapalogaster mertensii* (L; R), *Pagurus anachoretus* (L), *Phimochirus holthuisi* (L), *Probeebei mirabilis* (L; R), *Parapagurus pilosimanus* (L‐♀; L‐♂), *Sympagurus dimorphus* (L), *Xylopagurus cancellarius* (L).

**IV. scutiform chela (12 chelae; Figure** **6** **):** *Coenobita scaevola* (L), *Cryptolithodes sitchensis* (R), *Diogenes edwardsii* (L), *Dermaturus mandtii* (R), *Paguristes digueti* (L; R), *Phimochirus holtuisi* (R), *Diogenes pugilator* (L), *Parapagurus pilosimanus* (R‐♀; R‐♂), *Sympagurus dimorphus* (R), *Xylopagurus cancellarius* (R).

**V. discoid chela (7 chelae; Figure** **7** **):** *Agaricochirus erosus* (R), *Isocheles pilosus* (L; R), *Manucomplanus spinulosus* (R‐♀; R‐♂), *Pylopagurus discoidalis* (R‐d), *Pagurus beringanus* (R).

**VI. semidiscoid chela (4 chelae; Figure** **8** **):** *Bathycheles integer* (L; R), *Pomatocheles jeffreysii* (L; R).

The scatterplot of the shape types based on PCs 1 and 2 (explaining 57.48% of variance) is shown in Figure 9A; an overlap was evident for the shape types I, II, V, and VI. The LDA scatterplot (Figure 9B) revealed an overlap for shape type I (compact) and shape type II (sturdy). MANOVA demonstrated significant differences between all shape types, except for shape type VI (semidiscoid) (see Table 1A). Significant differences among shape types I, II, III, and IV were confirmed by PERMANOVA, whereas shape type V (discoid) exhibited a significant separation only from shape type VI (Table 1B). However, the results for shape type VI were considered as unreliable due to statistical artifacts (Zelditch et al. 2012). Another noteworthy detail was the lack of significant differences for shape type V (discoid) in PERMANOVA, despite a clear group separation in LDA (Figure 9B) and high *MD^2^* (Table 1A).

### 3.2 Chelae Shape Variation in a Functional and Systematic Context

**Figure 10:**
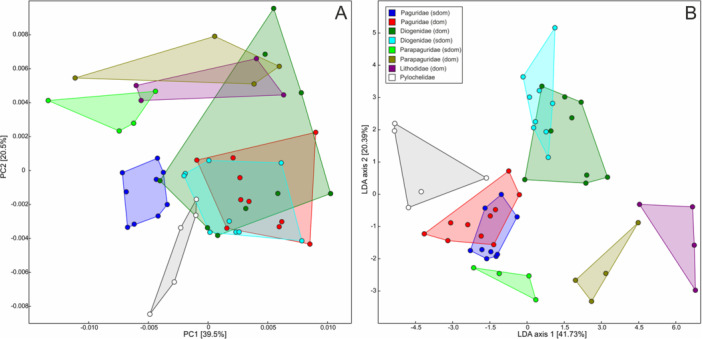
(A) Scatterplot of PC1 and PC2 and (B) scatterplot of LDA axes 1 and 2 of the data set for **test 1**, displaying the side‐specific classification at family level (54 chelae). According to LDA, 94.4% of chelae were correctly classified; classification accuracy decreased to 68.5% after jackknifing. Due to limitations in the sample size, following chela groups were excluded: Lithodidae (sdom), Xylopaguridae (dom) and (sdom), Coenobitidae (dom) and (sdom). Dom, dominant; sdom, subdominant.

**Test 1.** In the scatterplot of PCs 1 and 2 (Figure 10A), overlap was present for all groups based on side‐specific classification at family level. In the LDA scatterplot, the groups were closely positioned and exhibited partial overlap (Figure 10B). MANOVA did not reveal any significant separations between the groups (Supporting Information: Table S4A). In contrast, PERMANOVA found significant separations for the groups Pylochelidae, Lithodidae (dom), Parapaguridae (dom), and Parapaguridae (sdom) (Supporting Information: Table S4B). However, the corresponding *F*‐values for these significant separations were reported as *NAN*, which indicates statistical artifacts and limits the interpretability of the results. Given the statistical limitations and the observed results, a systematic signal in chela shape on this base is not detectable.

**Test 2.** No LDA scatterplot was generated due to the comparison of two groups. However, classification accuracy reached 91.1% (82.1% after JK). MANOVA and PERMANOVA found a significant separation of dominant and subdominant chelae across all examined left‐handed and right‐handed taxa (*p* < 0.001), with *F*‐values of 7.17 and 2.16, respectively.

**Figure 11:**
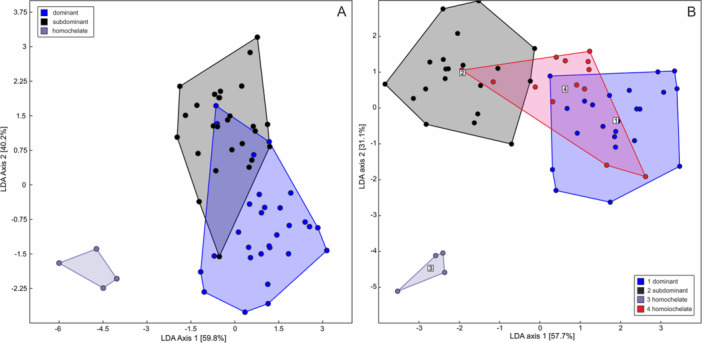
Scatterplots of LDA axes 1 and 2 showing the functional classification of **test 3** (A: dominant, subdominant, homochelate) and **test 4** (B: dominant, subdominant, homochelate, homoiochelate), based on 60 chelae (*T. boasi* was omitted). Numbers refer to the centroids of the four groups (B). LDA classification accuracy: test 3, 91.7% correctly classified (83.3% after JK); test 4, 90.2% correctly classified (72.1% after JK).

**Test 3.** For the third test (three groups), LDA found overlap between dominant and subdominant chelae and a distinct position of homochelate chelae (Figure 11A). MANOVA and PERMANOVA found significant separations between dominant, subdominant (representing both hetero‐ and homoiochelate chelae) and homochelate (semidiscoid) chelae (*p* < 0.05), with *F*‐values of 8.41 and 2.45, respectively.

**Test 4.** For the fourth test, the LDA scatterplot revealed an intermediate position of the homoiochelate chelae between the dominant and subdominant ones (Figure 11B), while homochelate (semidiscoid) chelae formed a distinct group. LDA assigned six homoiochelate chelae to the dominant and six to the subdominant group; however, this allocation included both left and right chelae being assigned to dominant and subdominant categories, despite the fact that in Diogenidae the dominant chela is the left one, while in Parapaguridae it is the right one (Supporting Information: Table S1). Based on the group centroid coordinates obtained from LDA, Euclidean distances were calculated, confirming the intermediate position of homoiochelate chelae and the distinct placement of homochelate chelae (Supporting Information S1: Table S5). The greatest distance, with 6.53, was found between homochelate and homoiochelate chelae. Multivariate analyses found significant separations between dominant, subdominant, and homochelate chelae (Table 2). MANOVA further revealed significant differences between homoiochelate chelae and both dominant and subdominant ones (Table 2A), while PERMANOVA did not (Table 2B). Taken together, these results illustrate consistent trends across different statistical approaches.

**Table 2:** Results of the MANOVA (A) and one‐way PERMANOVA (B) of the classification into subdominant, dominant, homoiochelate, and homochelate chelae (test 4). *F*‐values are colorized green, *p*‐values orange, and squared Mahalanobis distances (*MD^2^*) blue. Overall *F*‐values can be found in the top‐left cell. Significant differences between groups are marked with bold *p*‐values. *Fail* entries refer to insufficient sample size.

	subdominant	dominant	homoiochelate	homochelate
**A *F*: 8.416**				
subdominant	*p*‐values	**> 0.001**	**0.002**	**0.008**
dominant	15.858		**0.023**	**> 0.001**
homoiochelate	10.328	6.545		*fail*
homochelate	29.793	38.293	*fail*	*MD^2^*
**B *F*: 2.4**				
subdominant	*p*‐values	**> 0.001**	0.079	**0.014**
dominant	2.467		0.587	**0.004**
homoiochelate	1.538	1.332		1
homochelate	1.452	1.556	0.768	*F*‐values

In test **5A** we tested for side‐specific shape differences within dominant and subdominant chelae across left‐handed (Diogenidae and Coenobitidae) and right‐handed taxa (Paguridae, Parapaguridae, Lithodidae, and Xylopaguridae) excluding Pylochelidae. Significant separations were found, and LDA yielded high classification accuracy, also after jackknifing (Table 3.5A). The following analyses were done to narrow down and characterize these side‐specific morphological differences.

**Table 3:** Results of MANOVAs and one‐way PERMANOVAs testing for the side‐specific distinction of dominant and subdominant chelae (test 5). *F*‐values are colorized green, *p*‐values orange, significant differences between left and right chelae are marked with bold *p*‐values. LDA resulted in classification accuracy values of tested chelae. JK, jackknifed.

	Dominant chelae			Subdominant chelae		
Sample	MANOVA	PERMANOVA	LDA	MANOVA	PERMANOVA	LDA
**5A** all taxa excluding:	***p*** < **0.001**	***p*** < **0.001**	96.6%75.9% (JK)	***p*** < **0.001**	***p*** < **0.001**	100%
− Pylochelidae	*F* = 8.77	*F* = 1.59		*F* = 8.77	*F* = 1.57	84.6% (JK)
**5B** all taxa excluding:	***p*** < **0.001**	***p*** = **0.02**	100%	***p*** < **0.001**	***p*** = **0.001**	100%
− Pylochelidae − Lithodidae − Coenobitidae	*F* = 7.76	*F* = 1.4	79.2% (JK)	*F* = 7.76	*F* = 1.34	86.4% (JK)
**5C** all taxa excluding:	***p*** < **0.001**	*p* = 0.07	100%	***p*** < **0.001**	***p*** = **0.005**	100%
− Pylochelidae − Lithodidae − Parapaguridae − Coenobitidae	*F* = 8.74	*F* = 1.19	60% (JK)	*F* = 8.74	*F* = 1.09	94.4% (JK)

In test **5B** Pylochelidae, Lithodidae and Coenobitidae were excluded from the original taxon sampling. All multivariate tests yielded significant results, while LDA yielded high classification accuracy, also after JK (Table 3.5B).

In test **5C** Pylochelidae, Lithodidae, Coenobitidae, and Parapaguridae were excluded. All multivariate tests, except PERMANOVA for dominant chelae, showed significant separations (Table 3.5C). However, LDA classification accuracy for dominant chelae was relatively low after jackknifing (60%).

**Figure 12:**
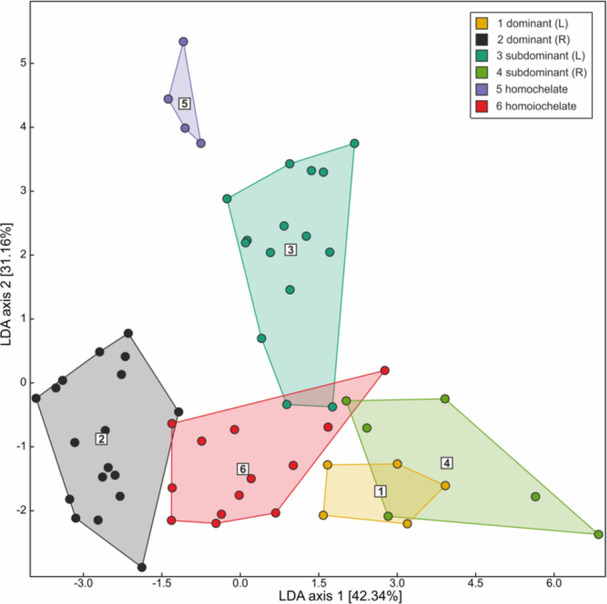
Scatterplot of LDA axes 1 and 2 of the original taxon sampling without *T. boasi* (**test 6**). Classification into dominant (left and right), subdominant (left and right), homochelate (semidiscoid) and homoiochelate chelae is depicted. Numbers 1 to 6 refer to the group centroids. LDA demonstrated a high classification accuracy of 95.1% (73.8% after JK).

**Test 6.** Finally, we examined the combination of tests 4 and 5. This way, the positions of homochelate (semidiscoid) and homoiochelate chelae in relation to the side‐specific dominance status—dominant and subdominant on the left and right sides—within heterochelate species could be analyzed (six groups). The distinct position of homochelate chelae is illustrated in the LDA scatterplot (Figure 12). Centroid distances regarding homochelate chelae ranged from 5.52 (subdominant (L) chelae) to 7.05 (homoiochelate chelae) (Supporting Information: Table S6). MANOVA significantly separated homochelate chelae from subdominant (L), dominant (R), and the homoiochelate ones, while the tests for subdominant (R) and dominant (L) failed (Table 4A). PERMANOVA significantly separated homochelate chelae from subdominant (L), dominant (R), subdominant (R), and dominant (L); however, separations involving subdominant (R) and dominant (L) also encountered statistical artifacts (Table 4B). Centroid distances to the homoiochelate chelae group ranged from 3.39 (dominant (R) chelae) to 7.05 (homochelate chelae) (Supporting Information: Table S6). MANOVA significantly separated homoiochelate chelae from all other groups except dominant (L) (Table 4A), whereas PERMANOVA found one significant separation between dominant (R) and homoiochelate chelae (Table 4B). Regarding homoiochelate chelae, the inconsistency of multivariate analyses, the low centroid distances to other groups (except to homochelate chelae), and their position in the LDA scatterplot (Figure 12) illustrate the intermediate position within the taxon sampling.

**Table 4:** Results of the MANOVA (A) and one‐way PERMANOVA (B) for test 6 with the classifications: homochelate, homoiochelate, subdominant (L), dominant (R), subdominant (R), and dominant (L). *F*‐values are colorized green, *p*‐values orange, and squared Mahalanobis distances (*MD^2^*) blue. Overall *F*‐values can be found in the top‐left cell. Significant differences between groups are marked with bold *p*‐values. *Fail* entries refer to insufficient sample size, while *NAN* (not a number) entries refer to mathematical artifacts. L, left; R, right; dom, dominant; sdom, subdominant.

	sdom (L)	dom (R)	homochelate	homoiochelate	sdom (R)	dom (L)
**A *F*: 13.96**
sdom (L)	*p*‐values	**< 0.001**	**0.007**	**< 0.001**	**< 0.001**	**0.007**
dom (R)	20.354		**< 0.001**	**< 0.001**	**< 0.001**	**< 0.001**
homochelate	26.495	35.287		**0.007**	*fail*	*fail*
homoiochelate	13.956	10.037	42.524		**0.029**	0.272
sdom (R)	24.81	44.204	*fail*	16.949		*fail*
dom (L)	20.012	27.192	*fail*	11.498	*fail*	*MD^2^*
**B *F*: 2.57**
sdom (L)	*p*‐values	**< 0.001**	**0.01**	0.129	**0.023**	0.093
dom (R)	2.733		**0.002**	**0.015**	**0.005**	**< 0.001**
homochelate	1.509	1.754		0.089	**< 0.001**	**< 0.001**
homoiochelate	1.693	1.973	1.263		1	1
sdom (R)	1.6	1.84	*NAN*	1.31		**< 0.001**
dom (L)	1.497	1.813	*NAN*	1.251	*NAN*	*F*‐values

## 4 Discussion

This study investigated chela shape variation across 30 paguroid species, aiming to characterize morphological patterns—expressed as chela shape types—and the three symmetry categories (heterochelate, homoiochelate, homochelate) in an evolutionary and functional context. Due to the substantial methodological effort required for detailed shape analyses using *SPHARM* (Ege et al. 2020, 2025), sample sizes per species were generally small, mostly represented by single individuals. Such small sample sizes might reduce statistical power and affect the interpretability of results, particularly for methods sensitive to within‐group variance, such as permutation‐based approaches (Anderson 2001); hence, statistical outcomes depend not only on morphological differentiation, but also on sample size and the statistical method applied (Zelditch et al. 2012). In small groups, a non‐significant result may therefore reflect either a true absence of effect or insufficient power to detect one. A significant result despite a small sample size, however, can generally be considered robust, if produced by the outlier‐robust Mahalanobis distance used in MANOVA and PERMANOVA (see Methods). Although some patterns appeared morphologically consistent, in some cases the small sample sizes precluded a robust statistical confirmation.

To account for the potential influence of shape‐dependent sexual dimorphism, we expanded the original taxon sampling by adding seven female and male individuals of *P. bernhardus*. Within this extended taxon sampling (see Supporting Information: Table S3A and Figure S1), sexual dimorphism for both dominant and subdominant chelae in *P. bernhardus* was not detectable. Similarly, in species for which both sexes were available (*M. spinulosus* and *P. pilosimanus*), our analyses did not show any sex‐specific separations. Minor sexual dimorphism has been described for *P. bernhardus* (Ege et al. 2025) as well as for *M. spinulosus* and *P. pilosimanus*, where notable shape differences occur particularly in the dominant (right) cheliped (Lemaitre 1986; Poore and Ahyong 2023). Because effective shell closure is essential for survival in both sexes, involved chelae must maintain a species‐specific morphology that ensures this function. As a result, any sex‐specific divergence in chela shape is therefore expected to be comparatively minor. This interpretation is consistent with our analyses; accordingly, sexual dimorphism was considered negligible for the present study and was not included as a factor.

### 4.1 Shape Types

We identified six distinct chela shape types. Shape types I (compact), II (sturdy), III (elongate) and IV (scutiform) were significantly distinguishable across statistical methods. The separation of shape type V (discoid) from the other shape types was statistically supported by MANOVA; in contrast, PERMANOVA did not detect a significant separation of shape type V, likely due to a combination of small sample size and increased within‐group variance through permutation (Anderson 2001). Although morphologically consistent, the small sample size precluded a robust conclusion. Hence, we consider the assignments of chelae to shape types V and VI correct, although their statistical robustness needs to be tested with a larger sample.

Shape types I (compact) and III (elongate) mostly comprise subdominant chelae, whereas shape types IV (scutiform) and V (discoid) are primarily composed of dominant chelae. This pattern is consistent with a functional signal: dominant chelae of the types IV and V show morphological features previously associated with shelter closure and defence (Magnus 1961; Reese 1969; Dowds and Elwood 1983; Elwood and Stewart 1985; Tudge et al. 2012), whereas chelae of shape types I and III exhibit features consistent with food manipulation and grooming (Mariappan et al. 2000). Shape type II (sturdy) shows no clear tendency with respect to hetero‐, homo‐, and homoiochely but includes the highest number of homoiochelate chelae, with five out of 14 sturdy chelae assigned to this symmetry category. However, homoiochelate chelae are distributed across all shape types except for type VI (semidiscoid), which comprises only homochelate chelae (Pylochelidae).

### 4.2 Testing of Symmetry Categories and Side‐Specific Differences

The side‐specific classification of chelae across hermit crab families (test 1) revealed no clear systematic differentiation. Even though LDA showed a high classification accuracy with only the dominant and subdominant groups of Diogenidae and Paguridae overlapping in the scatterplot.

We found a highly significant separation between dominant and subdominant chelae between all examined left‐handed and right‐handed (hetero‐ and homoiochelate) taxa (test 2). Furthermore, a distinct separation of homochelate chelae relative to both, dominant and subdominant chelae (including homoiochelate taxa), was statistically confirmed (test 3). A further subdivision of non‐homochelate chelae revealed an intermediate position of homoiochelate chelae between the heterochelate dominant and subdominant ones (test 4). Here, the greatest distance between group centroids within LDA was found for the homochelate and homoiochelate group. In test 5A significant side‐specific differences in the shape of dominant and subdominant chelae were found across all heterochelate and homoiochelate taxa (56 chelae). In the following tests 5B and 5C, taxa were sequentially excluded to test whether the observed side‐specific shape differences in dominant chelae are attributable to shell‐dwelling behavior. In test 5B, the non‐shell‐dwelling Lithodidae and the shell‐remodelling *Coenobita* (Laidre et al. 2012; Laidre 2019; Vermeij 2020) were excluded (47 chelae), which still yielded significant differences. In test 5C, Parapaguridae were additionally excluded (41 chelae), as their lifestyle often includes alternative defense strategies than shell‐dwelling. Here, the statistical analyses revealed inconsistent results for dominant chelae. Similar to test 5A, shape differences in subdominant chelae were still validated for both tests 5B and 5C. In test 6 (combining test 4 and 5A), homochelate chelae affirmed their distinct position relative to the other groups. In contrast, homoiochelate chelae exhibited only weak and inconsistent separations across multivariate analyses. Together with comparatively small centroid distances to the heterochelate dominant and subdominant groups and their intermediate position in the LDA scatterplot (similar to test 4), this suggests that homoiochelate chelae show a reduced degree of morphological differentiation compared to the other groups.

### 4.3 Form, Function, Phylogeny

Our findings suggest that the evolution of chela morphology in Paguroidea cannot be understood by considering phylogenetic history or functional demands in isolation. Instead, form, function, biological role (*sensu* Bock and von Wahlert 1965), and phylogenetic position must be seen as dynamically interacting forces leading to morphological changes (Lauder 1981). Selective pressures related to key biological roles (e.g., shelter closure, defense) presumably drive convergent morphological adaptations in chelae across “unrelated” taxa (i.e., left‐handed and right‐handed asymmetrical hermit crabs). Conversely, a conserved biological role exerts continuous selection pressure. The symmetrical, semidiscoid chelae of Pylochelidae apparently reflect this pattern, as their chela shape corresponds to a conserved biological role (shelter closure). However, any phylogenetic interpretation of chelae evolution in hermit crabs faces the difficulties of quite uncertain phylogenetic relationships.

### 4.4 Symmetry Categories (Heterochely, Homochely, Homoiochely) and Associated Functions/Biological Roles

Heterochelate taxa possess a dominant chela, which is distinctly larger and specialized for shelter closure (Magnus 1961; Reese 1969; Tudge et al. 2012), and a subdominant chela, usually smaller and shape‐wise less specialized, fulfilling different biological roles related to food manipulation, and grooming (Mariappan et al. 2000), Here, the cutting edge is more important for performance than the overall shape (Schäfer 1954). Homochelate taxa, in contrast, possess mirrored chelae of identical size and shape, correlating with biological roles that require both chelae jointly or that either chela can fulfill equivalently. Homoiochely represents an intermediate condition between these two conditions.

To understand the shape‐dependent differences in the dominant chelae of left‐handed and right‐handed hermit crabs, it is important to comprehend that gastropod shells, which serve as shelter for most heterochelate hermit crab taxa, are mainly dextral (Vermeij 1975; Tudge et al. 2012), as estimates have found up to 99% of living gastropods with right‐coiled shells (Asami 1993). This one‐sided ratio might explain the significant side‐specific differences in the shape of dominant chelae. The dorsal side of the dominant chela in right‐handed hermit crabs contacts the parietal wall of the shell aperture during closure, while the ventral side primarily contacts the palatal region. In left‐handed hermit crabs, this relationship is reversed, which may directly influence the side‐specific shape differences by creating opposing concave and convex “forces” on the dorsal and ventral sides of dominant left and right chelae (Figure 1B,C, dominant heterochelate chelae). In contrast, the side‐specific differences in subdominant chelae cannot be explained by shell closure, but rather reflect the independent evolutionary origins of heterochely in distinct lineages (Spani et al. 2020), as evolutionary trajectories are contingent on historical and random factors and therefore do not repeat identically (Blount et al. 2018). Nevertheless, the morphology of the aperture of the gastropod shell remains an important factor influencing the shape of dominant chelae in shell‐dwelling hermit crabs (Ege et al. 2025).

A subsequent relaxation of selective pressures related to shelter closure, driven by alternative defensive strategies, may have promoted homoiochely. In this context, homoiochelate taxa show reduced chela differentiation, placing them morphologically between dominant and subdominant heterochelate forms. Homoiochelate species (e.g., *Clibanarius erythropus, Ciliopagurus strigatus*) often prefer large gastropod shells that allow a deep withdrawal of the hermit crab (Peidro‐Devesa et al. 2025), or inhabit cone‐shells with a very narrow aperture (Hazlett 2009), making an operculiform closure less necessary. Consequently, in homoiochelate taxa, those functions, which in heterochelate taxa exhibit a strong link to the subdominant chela, presumably become more prominent influences on the morphology of both chelae, often resulting in shape type II (sturdy). At the same time, the comparatively large distance between homochelate and homoiochelate taxa in LDA morphospace, likely reflects their evolutionary distance.

### 4.5 Family‐Specific Variations in Chela Shape

The comparative analyses across hermit crab families reveal considerable morphological diversity in chela shape. While Pylochelidae show mostly clear clustering patterns, other families exhibit contra‐intuitive patterns, including an overlap between dominant and subdominant chelae (Paguridae and Diogendiae). Often, the patterns require a closer examination of the respective species, as specific ecological and morphological traits can obscure the general functional differentiation between dominant and subdominant chelae. Special adaptations are also present in the other families.

### 4.6 Paguridae

Within Paguridae, the cluster analysis revealed two distinct groups based on dominant chela shape. One group, including species such as *A. erosus*, *M. spinulosus*, and *P. holthuisi*, exhibits dominant chelae specialized for effective shell closure, corresponding to shape types IV (scutiform) and V (discoid). In contrast, the other group, represented by *P. bernhardus*, *P. cuanensis*, and *P. anachoretus*, displays a more generalized dominant chela shape (shape type II, sturdy), likely reflecting a broader range of shell species utilized. Consequently, the dominant chelae of the “*Pagurus‐*group” (shape type II, sturdy) and the subdominant pagurid chelae (mostly shape type I, compact) are morphologically more similar than the two dominant groups (scutiform and discoid vs. sturdy). Among extant Paguridae, *P. discoidalis* represents one of the most specialized “shelter closers”, as the occurrence of two different dominant chela morphs (Williams 1984; McLaughlin and Lemaitre 2001), allocated to shape types II (sturdy) and V (discoid) (*cf*. Ege et al. 2025) depending on shelter conditions (McLaughlin and Lemaitre 2001) likely reflects a functional specialization for round apertures, which resulted in the distinct chela shape type V (discoid). This might further contribute to the proximity between dominant and subdominant pagurid chelae within the LDA scatterplot.

### 4.7 Lithodidae

As representatives of Lithodidae, *Hapalogaster* does not use any kind of “hermit crab shelter”. This defensive strategy was secondarily lost (*cf*. “hermit to king” hypothesis – Boas 1924; Cunningham et al. 1992; Richter and Scholtz 1994), suggesting that other biological roles and functions than shelter closure show the primary influence on the shape of dominant chelae in Lithodidae. Here, *H. grebnitzkii* and *H. mertensii* exhibit dominant chelae assigned to shape type III (elongate), which otherwise comprises subdominant and homoiochelate chelae. Nevertheless, Lithodidae, which evolved from right‐handed hermit crabs (Cunningham et al. 1992; Richter and Scholtz 1994), exhibit pronounced heterochely, particularly in larger species (Poore and Ahyong 2023). This pronounced heterochely seems to persist despite the evolutionary transition to a free‐living lifestyle (*cf*. homoiochely in *P. mirabilis*). Heterochely might be crucial for other key biological roles in Lithodidae, especially food‐manipulating tasks and agonistic behavior, as large king and stone crab species show functional chela specialization similar to other large free‐living decapods. Lobsters and many brachyuran crabs show a functional differentiation between “crusher” and “cutter claws” (Przibram 1905; Schäfer 1954), although this pattern is generally less strictly side‐specific than in Lithodidae. In numerous heterochelate brachyuran populations, the dominant chela occurs predominantly on the right side, often in up to 80% of individuals. Left‐side dominance is typically interpreted as a consequence of autotomy and subsequent regeneration (Simonson 1985; Masunari et al. 2015). In Lithodidae, however, the strict side‐specificity probably simply reflects their evolutionary history. Smaller lithodids like *C. sitchensis* utilize their chelae in combination with a distinct carapace shape for the protection of their oral region, comparable to the defensive strategy characteristic for Calappidae (Ng and Tan 1985; Bellwood 2002). Consistent with this defensive role, and unlike *Hapalogaster*, *C. sitchensis* possesses a dominant chela of shape type IV (scutiform), despite being free‐living.

### 4.8 Diogenidae and Coenobitidae

Within Diogenidae, a basal scutiform shape of the dominant (left) chela is assumed, presumably functioning primarily in asymmetric shelter closure (*cf*. Paguridae). The suggested polyphyly of Diogenidae (Bracken‐Grissom et al. 2013), would imply the evolution of a left dominant chela for shell closure twice. Some diogenid lineages evolved alternative defensive strategies, such that specific morphological specializations (e.g., operculiform closure) in the dominant chela became less essential. Consequently, homoiochelate taxa are frequently found within Diogenidae (Poore and Ahyong 2023). This trend might have evolved independently in multiple taxa (e.g., *Clibanarius, Isocheles, Dardanus, Paguristes*) and is often associated with comparatively large gastropod shells enabling a complete retraction as alternative defensive strategy. In *Cancellus*, the level of chela symmetry has exceeded homoiochely, making them homochelate (Mayo 1973), which apparently is a condition correlated with a lifestyle and defense strategy similar to that of homochelate Pylochelidae. In the case of *Petrochirus*, this trend went even further, resulting in evolving a dominant right chela (Bertini and Fransozo 1999).

In the semiterrestrial Coenobitidae, the scutiform “ground pattern” of the dominant (left) chela has been retained, despite their ability to modify shells. The dominant chela, together with the second and third thoracopods (depending on shell conditions), form a tight operculum (Volker 1969). *Coenobita* actively collects and retains water in its shell (Gross 1964; De Wilde 1973; McMahon and Burggren 1979), a behavior that supports its semiterrestrial lifestyle. Because an effective shell closure is essential to avoid dehydration in warmer, mostly terrestrial (as adults) habitats, the operculum is particularly important. This is complemented by behavioral strategies such as nocturnal activity and refuge‐seeking during the day, which further reduce desiccation risk (Achituv and Ziskind 1985).

### 4.9 Parapaguridae

Parapaguridae present other intriguing cases. Right‐handed lineages (e.g., represented by *P. pilosimanus* and *S. dimorphus*) are inferred to ancestrally possess a dominant right chela of shape type IV (scutiform). Based on the supposed phylogenetic relationships, right‐dominant heterochely might have evolved convergently in Paguridae and Parapaguridae. In any case, different habitats with corresponding ecological demands have influenced their lifestyle and thus chela morphology. The scarcity of gastropod shells in the deep sea often promoted shifts in shelter use, including symbiosis with carcinoecium‐forming Anthozoa, leading to obligate ecological dependencies in many lineages (Muirhead et al. 1986; Williams and McDermott 2004; Crowther et al. 2011; Kise et al. 2019; Wright et al. 2020). These “pseudo‐shells” provide structural support in place of a gastropod shell, while the associated anthozoans offer nematocyst‐based defense. For example, *P. mirabilis* is a freely roaming species (Cuvelier et al. 2023), not carrying a shell; It exhibits a carcinized pleon, different from other Parapaguridae but similar to Lithodidae (Wolff 1961; Lemaitre 1998; Anker and Paulay 2013) but has also been observed carrying anemones on its pleon (Drazen et al. 2019; Christodoulou et al. 2022). As a result, selective pressures for operculiform dominant chelae declined, potentially facilitating the prioritization of other key biological roles in chela shape. The elongate (shape type III) chelae of *P. mirabilis*, for instance, are consistent with its ecological (scavenging) role and feeding behavior in the muddy deep sea (Bluhm 1994, 2001; Drazen et al. 2019).

### 4.10 Pylochelidae

The cluster analysis revealed that shape type VI (semidiscoid) is systematically confined to Pylochelidae, with homochelate chelae (McLaughlin and Lemaitre 2009; Tudge et al. 2012; Poore and Ahyong 2023). Forest (1987a, 1987b) documented that different pylochelid taxa utilize different types of shelters, correlating with their habitat. For example, wood‐dwelling (xylicolous) species are regularly found near forested islands where wood debris is common. *Pomatocheles*, which is petricolous and dwells primarily on soft stones or pumice pebbles, is typically associated with volcanic regions (Poore and Ahyong 2023). Pylochelinae, including *B. integer*, are xylicolous and inhabit wood debris in deep‐sea channels (Poore and Ahyong 2023). However, both, *P. jeffreysii* and *B. integer* exhibit semidiscoid (*cf*. Forest 1987a—operculiform) chelae (shape type VI) presumably adapted for symmetrical shelter closure apparently independent of the nature of their shelter. *Trizocheles boasi* stands out from this pattern. Its chela was assigned to shape type II (sturdy) which morphologically and functionally excludes *T. boasi* from the (symmetrical) semidiscoid category and contrasts with most Pylochelidae. This is consistent with previous observations indicating that chelae of Trizochelinae are non‐operculiform (Forest 1987a). Trizochelinae are spongicolous, and mostly inhabit thin‐walled tubular sponges such as dictyonin hexactinellids (Forest 1987b; Komai 2013). This kind of shelter reduces the need to form an operculiform closure and is consistent with their sturdy chela shape. Furthermore, Trizochelinae can exhibit homoiochelate or even heterochelate chelipeds (McLaughlin and Lemaitre 2009).

Regardless of whether symmetrical Pylochelidae are considered as sister group to all remaining asymmetrical hermit crabs (Richter and Scholtz 1994; Bracken‐Grissom et al. 2013) or as separate anomuran lineage (Tsang et al. 2011), their semidiscoid (shape type VI), homochelate chelae likely evolved in relation to their special case of shelter. This semidiscoid shape can be interpreted in two ways: it may provide a convincing argument for their monophyly (with Trizochelinae derived), or it may represent the plesiomorphic condition for the entire Paguroidea, as indicated by the fossil record (Schweitzer et al. 2009; Fraaije et al. 2013).

## 5 Conclusion

Six distinct chela shape types were identified across Paguroidea. Shelter closure is assumed as the key selective force shaping operculiform chelae (shape types IV—scutiform; V—discoid) in both left‐handed and right‐handed heterochelate lineages, whereas paired semidiscoid chelae (shape type VI) in homochelate taxa likewise reflect selection for shelter closure by functioning together as an operculum. The homoiochelate condition represents an independent state between asymmetric (heterochely) and fully symmetric chelae (homochely), reflecting reduced functional specialization. Homoiochely is frequently associated with alternative defensive strategies, such as reliance on narrow shell apertures, deep (complete) withdrawal into the shell, symbiosis with anthozoans, or a shift to external armor as in Lithodidae. In these lineages, relaxation of selection for operculiform closure enables chela morphology to diversify, favoring other biological roles, particularly food manipulation. Our findings illustrate how shifts in ecological demands can redirect morphological evolution beyond the constraints of phylogenetic history.

## Author Contributions

**Yannic C. Ege:** writing – original draft, writing – review and editing, visualization, conceptualization, investigation, formal analysis, data curation, software, methodology. **Christian Foth:** conceptualization, investigation, software, writing – review and editing, methodology, validation. **Stefan Richter:** supervision, project administration, writing – review and editing, funding acquisition, conceptualization, methodology, investigation, validation; resources.

## Supporting information


**Figure S1:** Ward's cluster analysis of the extended taxon sampling containing 7 additional female (blue) and male (red) specimens of *P. bernhardus*. The analysis is based on the first 12 PCs that account for over 95% of the variance of the PCA of the extended taxon sampling (see Table S3A). No sex‐specific shape differences for the dominant and subdominant chelae in *P. bernhardus* are depicted. Furthermore, MANOVA and PERMANOVA yield no significant separation between female and male chelae (*p* = 1), approving the results of the cluster analysis.

**Table S1:** Taxa included in the shape analyses and the side‐specific classification of their chelae into different shape types based on Ward's cluster analysis. Shape types are defined as follows: Type I – compact; type II – sturdy; type III – elongate; type IV – scutiform; type V – discoid; type VI – semidiscoid. F, female; m, male.
**Table S2:** Overview of the statistical test designs (1 to 6) conducted to investigate how chela shape reflects functional and systematic classifications. Each test is defined by its classification criterion, from which the analyzed groups and the effective sample size (after exclusions) are derived. The corresponding research aim specifies the analytical focus of each test, addressing morphological variation in relation to side‐specific dominance, symmetry category (homo‐, homoio‐, and heterochelate), ecological context, and systematic groupings. L, left; R, right.
**Table S3:** Summary of the PCs of the conducted PCA of the extended (A) and original taxon sampling (B). The extended taxon sampling includes seven female and seven male *P. bernhardus* specimens to test for shape‐dependent sexual dimorphism (see Figure S1). Orange cells mark PCs that together account for at least 95% of the total variance.
**Table S4:** Results of the MANOVA (A) and one‐way PERMANOVA (B) of the side‐specific classification at family level (**test 1**). *F*‐values are colorized green, *p*‐values orange, and squared Mahalanobis distances (*MD²*) blue. *Fail* entries refer to insufficient sample size, while *NAN* (not a number) refers to mathematical artifacts resulting from non‐normally distributed data groups. Significant differences between groups are marked with bold *p*‐values. Pag, Paguridae; Pylo, Pylochelidae; Dio, Diogenidae; Litho, Lithodidae; Para, Parapaguridae; L, left; R, right.
**Table S5:** Euclidean distances between group centroids of dominant, subdominant, homochelate, and homoiochelate chelae based on LDA (**test 4**). The longest distance is marked in bold.
**Table S6:** Euclidean distances between group centroids of heterochelate dominant left and right, heterochelate subdominant left and right, homochelate, and homoiochelate chelae based on LDA (**test 6**). L, left; R, right.

## Data Availability

The data that support the findings of this study are available on request from the corresponding author. The data are not publicly available due to privacy or ethical restrictions.

## References

[jmor70161-bib-0001] Achituv, Y. , and M. Ziskind . 1985. “Adaptation of *Coenobita scaevola* (Forskal) (Crustacea, Anemura) to Terrestrial Life in Desert‐Bordered Shore Line.” Marine Ecology Progress Series 25: 189–197.

[jmor70161-bib-0002] Ahyong, S. T. , and D. O'Meally . 2004. “Phylogeny of the Decapoda Reptantia: Resolution Using Three Molecular Loci and Morphology.” Raffles Bulletin of Zoology 52: 673–693.

[jmor70161-bib-0003] Ahyong, S. T. , K. E. Schnabel , and E. W. Maas . 2009. “Anomuran phylogeny: New Insights From Molecular Data.” In Crustacean Issues: Decapod Crustacean Phylogenetics, edited by J. W. Martin , K. A. Crandall , and D. L. Felder , 399–414. A.A. Balkema.

[jmor70161-bib-0004] Almón, B. , J. E. García‐Raso , and J. A. Cuesta . 2025. “A Multilevel Biodiversity Approach of the Hermit Crabs in the Iberian Peninsula and Ultraperipheral Territories.” Marine Ecology 46: e70011. 10.1111/maec.70011.

[jmor70161-bib-0005] Anderson, M. J. 2001. “Permutation Tests for Univariate or Multivariate Analysis of Variance and Regression.” Canadian Journal of Fisheries and Aquatic Sciences 58: 626–639.

[jmor70161-bib-0006] Anker, A. , and G. Paulay . 2013. “A Remarkable New Crab‐Like Hermit Crab (Decapoda: Paguridae) From French Polynesia, With Comments on Carcinization in the Anomura.” Zootaxa 3722: 293–300. 26171527

[jmor70161-bib-0007] Asami, T. 1993. “Genetic Variation and Evolution of Coiling Chirality in Snails.” Forma 8: 263–276.

[jmor70161-bib-0008] Bellwood, O. 2002. “Systematics, Biogeography and Functional Morphology of the Box Crabs (Family Calappidae)” Doctoral dissertation, James Cook University.

[jmor70161-bib-0009] Bertini, G. , and A. Fransozo . 1999. “Relative Growth of *Petrochirus diogenes* (Linnaeus, 1758) (Crustacea, Anomura, Diogenidae) in the Ubatuba Region, São Paulo, Brazil.” Revista Brasileira de Biologia 59: 617–625. 23505650 10.1590/s0034-71081999000400011

[jmor70161-bib-0010] Blount, Z. D. , R. E. Lenski , and J. B. Losos . 2018. “Contingency and Determinism in Evolution: Replaying Life's Tape.” Science 362: eaam5979. 10.1126/science.aam5979. 30409860

[jmor70161-bib-0011] Bluhm, H. 1994. “Monitoring Megabenthic Communities in Abyssal Manganese Nodule Sites of the East Pacific Ocean in Association With Commercial Deep‐Sea Mining.” Aquatic Conservation: Marine and Freshwater Ecosystems 4: 187–201.

[jmor70161-bib-0012] Bluhm, H. 2001. “Re‐Establishment of an Abyssal Megabenthic Community After Experimental Physical Disturbance of the Seafloor.” Deep Sea Research Part II: Topical Studies in Oceanography 48: 3841–3868.

[jmor70161-bib-0013] Boas, J. E. V. 1924. “Die Verwandtschaftliche Stellung Der Gattung *Lithodes* .” Det Kongelige Danske Videnskabernes Selskab, Biologiske Meddelelser 4: 1–34.

[jmor70161-bib-0014] Bock, W. J. , and G. von Wahlert . 1965. “Adaptation and the Form‐Function Complex.” Evolution 19: 269–299.

[jmor70161-bib-0015] Bracken‐Grissom, H. D. , M. E. Cannon , P. Cabezas , et al. 2013. “A Comprehensive and Integrative Reconstruction of Evolutionary History for Anomura (Crustacea: Decapoda).” BMC Evolutionary Biology 13: 128. 23786343 10.1186/1471-2148-13-128 PMC3708748

[jmor70161-bib-0016] Christodoulou, M. , S. De Grave , Α Vink , and P. Martinez Arbizu . 2022. “Taxonomic Assessment of Deep‐Sea Decapod Crustaceans Collected From Polymetallic Nodule Fields of the East Pacific Ocean Using an Integrative Approach.” Marine Biodiversity 52: 61.

[jmor70161-bib-0017] Cignoni, P. , M. Callieri , M. Corsini , M. Dellepiane , F. Ganovelli , and G. Ranzuglia . 2008. “MeshLab: An Open‐Source Mesh Processing Tool.” Eurographics Italian Chapter Conference 2008: 129–136.

[jmor70161-bib-0018] Crowther, A. , D. Fautin , and C. Wallace . 2011. “ *Stylobates birtlesi* sp. n., a new species of carcinoecium forming sea anemone (Cnidaria, Actiniaria, Actiniidae) From eastern Australia.” ZooKeys 89: 33–48. 10.3897/zookeys.89.825 PMC3082962 21594082

[jmor70161-bib-0019] Cunningham, C. W. , N. W. Blackstone , and L. W. Buss . 1992. “Evolution of King Crabs From Hermit‐Crab Ancestors.” Nature 355: 539–542. 1741031 10.1038/355539a0

[jmor70161-bib-0020] Cuvelier, D. , M. Vigneron , A. Colaço , and J. Greinert . 2023. “Delayed Response of Hermit Crabs Carrying Anemones to a Benthic Impact Experiment at the Deep‐Sea Nodule Fields of the Peru Basin?” Marine Environmental Research 185: 105899. 10.1016/j.marenvres.2023.105899. 36716607

[jmor70161-bib-0036] De Grave, S. , N. D. Pentcheff , S. T. Ahyong , et al. 2009. “A Classification of Living and Fossil Genera of Decapod Crustaceans.” Raffles Bulletin of Zoology 21: 1–109.

[jmor70161-bib-0099] De Wilde, P. A. W. 1973. “On the Ecology of *Coenobita clypeatus* in Curacao.” Studies on the Fauna of Curacao and other Caribbean Islands 44: 1–138.

[jmor70161-bib-0022] Dowds, B. M. , and R. W. Elwood . 1983. “Shell Wars: Assessment Strategies and the Timing of Decisions in Hermit Crab Shell Fights.” Behaviour 85: 1–24.

[jmor70161-bib-0023] Drazen, J. C. , A. B. Leitner , S. Morningstar , Y. Marcon , J. Greinert , and A. Purser . 2019. “Observations of Deep‐Sea Fishes and Mobile Scavengers From the Abyssal DISCOL Experimental Mining Area.” Biogeosciences 16: 3133–3146.

[jmor70161-bib-0024] Edmonds, E. , and M. Briffa . 2016. “Weak Rappers Rock More: Hermit Crabs Assess Their Own Agonistic Behaviour.” Biology Letters 12: 20150884. 10.1098/rsbl.2015.0884. 26740563 PMC4785928

[jmor70161-bib-0026] Ege, Y. C. , C. Foth , C. S. Wirkner , and S. Richter . 2025. “Asymmetrical Chela Growth Patterns and Correlation of Chela Shape to Inhabited Gastropod Shells in *Pagurus bernhardus* (Linnaeus, 1758) (Decapoda, Anomura, Paguridae).” Zoomorphology 144: 22.

[jmor70161-bib-0025] Ege, Y. C. , C. Foth , D. Baum , C. S. Wirkner , and S. Richter . 2020. “Adapting Spherical‐Harmonics‐Based Geometric Morphometrics (SPHARM) for 3D Images Containing Large Cavity Openings Using Ambient Occlusion: A Study With Hermit Crab Claw Shape Variability.” Zoomorphology 139: 421–432.

[jmor70161-bib-0027] Elwood, R. W. , and A. Stewart . 1985. “The Timing of Decisions During Shell Investigation by the Hermit Crab, *Pagurus bernhardus* .” Animal Behaviour 33: 620–627.

[jmor70161-bib-0028] Etherington, T. R. 2021. “Mahalanobis Distances for Ecological Niche Modelling and Outlier Detection: Implications of Sample Size, Error, and Bias for Selecting and Parameterising a Multivariate Location and Scatter Method.” PeerJ 9: e11436. 10.7717/peerj.11436. 34026369 PMC8121071

[jmor70161-bib-0029] Everitt, B. , and T. Hothorn . 2011. An Introduction to Applied Multivariate Analysis With R. Springer.

[jmor70161-bib-0030] Forest, J. 1987a. “ *Les Pylochelidae ou “Pagures symetriques” (Crustacea Coenobitoidea)*. Mémoires du Muséum National d'Histoire Naturelle.” Série A, Zoologie 137: 1–249.

[jmor70161-bib-0031] Forest, J. 1987b. “Ethology and Distribution of Pylochelidae (Crustacea Decapoda Coenobitoidea).” Bulletin of Marine Science 41: 309–321.

[jmor70161-bib-0032] Foth, C. , S. W. Evers , W. G. Joyce , V. S. Volpato , and R. B. J. Benson . 2019. “Comparative Analysis of the Shape and Size of the Middle Ear Cavity of Turtles Reveals No Correlation With Habitat Ecology.” Journal of Anatomy 235: 1078–1097. 31373396 10.1111/joa.13071 PMC6875938

[jmor70161-bib-0033] Fraaije, R. H. B. , B. W. M. van Bakel , J. W. M. Jagt , et al. 2022. “The Evolution of Hermit Crabs (Crustacea, Decapoda, Anomura, Paguroidea) on the Basis of Carapace Morphology: A State‐of‐the‐Art‐Report.” Geodiversitas 44: 1–16.

[jmor70161-bib-0085] Fraaije, R. , G. Schweigert , A. Nützel , and P. Havlik . 2013. “New Early Jurassic Hermit Crabs From Germany and France.” Journal of Crustacean Biology 33: 802–817.

[jmor70161-bib-0034] Gašparič, R. , R. H. B. Fraaije , N. Robin , and A. De Angeli . 2016. “The First Record of Paguroids From the Eocene of Istria (Croatia) and further Phylogenetic Refinement of the Paguroidea (Crustacea, Anomura).” Bulletin of Geosciences 91: 467–480.

[jmor70161-bib-0035] Gong, L. , X. Lu , Z. Wang , et al. 2020. “Novel Gene Rearrangement in the Mitochondrial Genome of *Coenobita brevimanus* (Anomura: Coenobitidae) and Phylogenetic Implications for Anomura.” Genomics 112: 1804–1812. 31655177 10.1016/j.ygeno.2019.10.012

[jmor70161-bib-0037] Gross, W. J. 1964. “Water Balance in Anomuran Land Crabs on a Dry Atoll.” Biological Bulletin 126: 54–68.

[jmor70161-bib-0038] Gruner, H. E. , M. Moritz , and W. Dunger . 1993. “3. Ordnung Decapoda, Zehnfußkrebse.” In Lehrbuch der speziellen Zoologie (begrundet von Alfred Kaestner), Band I: Wirbellose Tiere, 4. Teil: Arthropoda (ohne Insecta) (4. Auflage), 925–1030. Gustav Fischer Verlag.

[jmor70161-bib-0039] Hammer, O. , and D. A. T. Harper . 2006. Paleontological Data Analysis. Oxford, Blackwell.

[jmor70161-bib-0040] Hammer, O. , D. A. T. Harper , and P. D. Ryan . 2001. “PAST: Paleontological Statistics Software Package for Education and Data Analysis.” Palaeontologia Electronica 4: 1–9.

[jmor70161-bib-0042] Hazlett, B. 2009. “Notes on the Social Behavior of Some Hawaiian Hermit Crabs (Decapoda, Anomura).” Crustaceana 82: 763–768.

[jmor70161-bib-0041] Hazlett, B. A. 1974. “Field Observations on Interspecific Agonistic Behavior in Hermit Crabs.” Crustaceana 26: 133–138.

[jmor70161-bib-0043] Imafuku, M. 1983. “New Shell Acquisition in the Hermit Crab, Pagurus Geminus.” Journal of Ethology 1: 91–100.

[jmor70161-bib-0044] Jackson, D. A. 1993. “Stopping Rules in Principal Components Analysis: A Comparison of Heuristical and Statistical Approaches.” Ecology 74: 2204–2214.

[jmor70161-bib-0045] Keiler, J. , S. Richter , and C. S. Wirkner . 2015. “The Anatomy of the King Crab *Hapalogaster mertensii* Brandt, 1850 (Anomura: Paguroidea: Hapalogastridae)—New Insights Into the Evolutionary Transformation of Hermit Crabs into King Crabs.” Contributions to Zoology 84: 149–165.

[jmor70161-bib-0046] Kise, H. , J. Montenegro , M. Ekins , T. Moritaki , and J. D. Reimer . 2019. “A Molecular Phylogeny of Carcinoecium‐Forming *Epizoanthus* (Hexacorallia: Zoantharia) From the Western Pacific Ocean With Descriptions of Three New Species.” Systematics and Biodiversity 17: 773–786.

[jmor70161-bib-0047] Komai, T. 2013. “Records of Four Species of the Pylochelid Hermit Crab Genus *Trizocheles* Forest, 1987 (Crustacea: Decapoda: Anomura) From the Sagami Sea and Izu Islands, Central Japan, With Descriptions of Three New Species.” Natural History Research 12: 91–112.

[jmor70161-bib-0048] Laidre, M. E. 2012a. “Niche Construction Drives Social Dependence in Hermit Crabs.” Current Biology 22: 861–863. 10.1016/j.cub.2012.08.056 23098589

[jmor70161-bib-0049] Laidre, M. E. 2012b. “Homes for Hermits: Temporal, Spatial and Structural Dynamics as Transportable Homes Are Incorporated Into a Population.” Journal of Zoology 288: 33–40.

[jmor70161-bib-0050] Laidre, M. E. 2019. “Architectural Modification of Shells by Terrestrial Hermit Crabs Alters Social Dynamics in Later Generations.” Ecology 100: e02767. https://www.jstor.org/stable/26785431. 31162638 10.1002/ecy.2767

[jmor70161-bib-0051] Laidre, M. E. , and R. W. Elwood . 2008. “Motivation Matters: Cheliped Extension Displays in the Hermit Crab, *Pagurus bernhardus*, Are Honest Signals of Hunger.” Animal Behaviour 75: 2041–2047.

[jmor70161-bib-0052] Laidre, M. E. , E. Patten , and L. Pruitt . 2012. “Costs of a More Spacious Home After Remodelling by Hermit Crabs.” Journal of the Royal Society Interface 9: 3574–3577. 22896569 10.1098/rsif.2012.0501 PMC3481588

[jmor70161-bib-0053] Lauder, G. V. 1981. “Form and Function: Structural Analysis in Evolutionary Morphology.” Paleobiology 7: 430–442.

[jmor70161-bib-0054] Lemaitre, R. 1986. “Western Atlantic Species of the *Parapagurus pilosimanus* Complex (Anomura: Paguroidea: Parapaguridae): Description of a New Species and Morphological Variations.” Journal of Crustacean Biology 6: 525–542.

[jmor70161-bib-0055] Lemaitre, R. 1998. “Revisiting *Tylaspis anomala* Henderson, 1885 (Parapaguridae), With Comments on Its Relationships and Evolution.” Zoosystema 20: 289–305.

[jmor70161-bib-0056] Lemaitre, R. , and P. A. McLaughlin . 2009. “Recent Advances and Conflicts in Concepts of Anomuran Phylogeny (Crustacea: Malacostraca).” Arthropod Systematics & Phylogeny 67: 119–135.

[jmor70161-bib-0057] Magnus, D. B. E. 1961. “Zur Ökologie Des Landeinsiedlers *Coenobita Jousseaumei* Bouvier Und Der Krabbe *Ocypode aegiptiaca* Geestackner Am Roten Meer.” Zoologischer Anzeiger 38: 316–329.

[jmor70161-bib-0058] Mahalanobis, P. C. 1936. “On the Generalised Distance in Statistics.” Proceedings of the National Institute of Sciences India 2: 49–55.

[jmor70161-bib-0059] Mariappan, P. , C. Balasundaram , and B. Schmitz . 2000. “Decapod Crustacean Chelipeds: An Overview.” Journal of Biosciences 25: 301–313. 11022233 10.1007/BF02703939

[jmor70161-bib-0060] Masunari, N. , M. Hiro‐oku , S. Dan , et al. 2015. “Chela Asymmetry in a Durophagous Crab: Predominance of Right‐Handedness and Handedness Reversal Is Linked to Chela Size and Closing Force.” Journal of Experimental Biology 218: 3658–3670. 26417016 10.1242/jeb.120196

[jmor70161-bib-0107] Mayo, B. 1973. "A Review of the Genus Cancellus (Crustacea: Diogenidae) With the Description of a new Species From the Caribbean Sea." Smithsonian Contributions to Zoology 150: 1–63.

[jmor70161-bib-0061] McLaughlin, P. A. 2003. “Illustrated Keys to Families and Genera of the Superfamily Paguroidea (Crustacea: Decapoda: Anomura), With Diagnoses of Genera of Paguridae.” Memoirs of Museum Victoria 60: 111–144.

[jmor70161-bib-0062] McLaughlin, P. A. , and R. Lemaitre . 2001. “Revision of *Pylopagurus* and *Tomopaguru*s (Crustacea: Decapoda: Paguridae), With Descriptions of New Genera and Species. Part VI. *Pylopagurus* Milne‐Edwards and Bouvier, *Haigia* Mclaughlin, and *Pylopaguridium* New Genus.” Proceedings of the Biological Society of Washington 114: 444–483.

[jmor70161-bib-0063] McLaughlin, P. A. , and R. Lemaitre . 2009. “A New Classification for the Pylochelidae (Decapoda: Anomura: Paguroidea) and Descriptions of New Taxa.” Raffles Bulletin of Zoology 20: 159–231.

[jmor70161-bib-0066] McLaughlin, P. A. , and T. Murray . 1990. “ *Clibanarius fonticola*, New Species (Anomura: Paguridea: Diogenidae), From a Fresh‐Water Pool on Espiritu Santo, Vanuatu.” Journal of Crustacean Biology 10: 695–702.

[jmor70161-bib-0064] McLaughlin, P. A. , R. Lemaitre , and K. A. Crandall . 2010. “Annotated Checklist of Anomuran Decapod Crustaceans of the World (Exclusive of the Kiwaoidea and Families Chirostylidae and Galatheidae of the Galatheoidea) Part III—Aegloidea.” Raffles Bulletin of Zoology 23: 131–137.

[jmor70161-bib-0065] McLaughlin, P. A. , R. Lemaitre , and U. Sorhannus . 2007. “Hermit Crab Phylogeny: A Reappraisal and Its “Fall‐Out.” Journal of Crustacean Biology 27: 97–115.

[jmor70161-bib-0067] McMahon, B. R. , and W. W. Burggren . 1979. “Respiration and Adaptation to the Terrestrial Habitat in the Land Hermit Crab *Coenobita clypeatus* .” Journal of Experimental Biology 79: 265–281.

[jmor70161-bib-0068] Mitteroecker, P. , and F. Bookstein . 2011. “Linear Discrimination, Ordination, and the Visualization of Selection Gradients in Modern Morphometrics.” Evolutionary Biology 38: 100–114.

[jmor70161-bib-0069] Muirhead, A. , P. A. Tyler , and M. H. Thurston . 1986. “Reproductive Biology and Growth of the Genus Epizoanthus (Zoanthidea) From the Northeast Atlantic.” Journal of the Marine Biological Association of the United Kingdom 66: 131–143.

[jmor70161-bib-0070] Nascimento, W. M. , A. O. Almeida , and A. P. Pinheiro . 2024. “Shape Variation in the Snapping Claw of *Alpheus* Fabricius, 1898 (Decapoda: Alpheidae): A Geometric Morphometrics Approach.” Acta Zoologica 106: 195–204.

[jmor70161-bib-0071] Ng, P. K. L. , and L. W. H. Tan . 1985. “‘Right Handedness’ in Heterochelous Calappoid and Xanthoid Crabs—Suggestion for a Functional Advantage.” Crustaceana 49: 98–100.

[jmor70161-bib-0072] Osawa, M. , and Y. Fujita . 2008. “ *Clibanarius ambonensis* (Crustacea: Decapoda: Anomura: Diogenidae) From the Ryukyu Islands, South‐western Japan.” Marine Biodiversity Records 1: e16.

[jmor70161-bib-0073] Peidro‐Devesa, M. J. , J. Faria , A. C. Costa , A. Z. Botelho , and G. M. Martins . 2025. “Shifts in Patterns of Shell Utilization by the Hermit Crab *Clibanarius erythropus* Following the Arrival of a Non‐Native Gastropod.” Marine Environmental Research 205: 107004. 10.1016/j.marenvres.2025.107004. 39952221

[jmor70161-bib-0074] Poore, G. C. , and S. T. Ahyong . 2023. Marine Decapod Crustacea: A Guide to Families and Genera of the World. Commonwealth Scientific and Industrial Research Organisation.

[jmor70161-bib-0075] Przibram, H. 1905. “Die »Heterochelie« Bei Decapoden Crustaceen (Zugleich: Experimentelle Studien Über Regeneration. Dritte Mitteilung).” Archiv für Entwicklungsmechanik der Organismen 19: 181–247.

[jmor70161-bib-0076] Przibram, H. 1907. “Differenzierung Des Abdomens Enthäuster Einsiedlerkrebse (Paguridae).” Archiv für Entwicklungsmechanik der Organismen 23: 579–595.

[jmor70161-bib-0077] Reese, E. S. 1962. “Shell Selection Behaviour of Hermit Crabs.” Animal Behaviour 10: 347–360.

[jmor70161-bib-0078] Reese, E. S. 1969. “Behavioral Adaptations of Intertidal Hermit Grabs.” American Zoologist 9: 343–355.

[jmor70161-bib-0079] Reimann, A. , S. Richter , and G. Scholtz . 2011. “Phylogeny of the Anomala (Crustacea, Decapoda, Reptantia) Based on the Ossicles of the Foregut.” Zoologischer Anzeiger—A Journal of Comparative Zoology 250: 316–342.

[jmor70161-bib-0080] Richter, S. , and G. Scholtz . 1994. “Morphological Evidence for a Hermit Crab Ancestry of Lithodids (Crustacea, Decapoda, Anomala, Paguroidea).” Zoologischer Anzeiger 233: 187–210.

[jmor70161-bib-0081] Schäfer, W. 1954. “Form Und Funktion Der Brachyuren‐Schere.” Abhandlungen der Senckenbergischen naturforschenden Gesellschaft 489: 1–65.

[jmor70161-bib-0082] Schembri, P. J. 1982. “Feeding Behaviour of Fifteen Species of Hermit Crabs (Crustacea: Decapoda: Anomura) From the Otago Region, Southeastern New Zealand.” Journal of Natural History 16: 859–878.

[jmor70161-bib-0084] Scholtz, G. 1995. “Phylogenetic Systematics of the Reptantian Decapoda (Crustacea Malacostraca).” Zoological Journal of the Linnean Society 113: 289–328.

[jmor70161-bib-0083] Scholtz, G. 2014. “Evolution of Crabs – History and Deconstruction of a Prime Example of Convergence.” Contributions to Zoology 83: 87–105.

[jmor70161-bib-0021] Schram, F. , S. Ahyong , and C. Dixon . 2003. “A New Hypothesis of Decapod Phylogeny.” Crustaceana 76: 935–975.

[jmor70161-bib-0086] Schweitzer, C. E. , R. M. Feldmann , and I. Lazǎr . 2009. “Fossil Crustacea (Excluding Cirripedia and Ostracoda) in the University of Bucharest Collections, Romania, Including Two New Species.” Bulletin of the Mizunami Fossil Museum 35: 1–14.

[jmor70161-bib-0088] Shen, L. , and F. Makedon . 2006. “Spherical Mapping for Processing of 3D Closed Surfaces.” Image and Vision Computing 24: 743–761.

[jmor70161-bib-0087] Shen, L. , H. Farid , and M. A. McPeek . 2009. “Modeling Three‐Dimensional Morphological Structures Using Spherical Harmonics.” Evolution 63: 1003–1016. 19154365 10.1111/j.1558-5646.2008.00557.x PMC2936781

[jmor70161-bib-0089] Silva, I. C. , and J. Paula . 2008. “Is There a Better Chela to Use for Geometric Morphometric Differentiation in Brachyuran Crabs? A Case Study Using *Pachygrapsus marmoratus* and *Carcinus maenas* .” Journal of the Marine Biological Association of the United Kingdom 88: 941–953.

[jmor70161-bib-0090] Simanjuntak, R. G. , and R. Eprilurahman . 2019. “Geometric Morphometrics Analysis of Chelae and Carapace of the Freshwater Prawn *Macrobrachium* Bate, 1868.” Biogenesis: Jurnal Ilmiah Biologi 7: 58–66.

[jmor70161-bib-0091] Simonson, J. L. 1985. “Reversal of Handedness, Growth, and Claw Stridulatory Patterns in the Stone Crab *Menippe mercenaria* (Say) (Crustacea: Xanthidae).” Journal of Crustacean Biology 5: 281–293.

[jmor70161-bib-0092] Spani, F. , M. Scalici , K. A. Crandall , and P. Piras . 2020. “Claw Asymmetry in Crabs: Approaching an Old Issue From a New Point of View.” Biological Journal of the Linnean Society 129: 162–176.

[jmor70161-bib-0093] Tsang, L. M. , T. Y. Chan , S. T. Ahyong , and K. H. Chu . 2011. “Hermit to King, or Hermit to All: Multiple Transitions to Crab‐Like Forms From Hermit Crab Ancestors.” Systematic Biology 60: 616–629. 21835822 10.1093/sysbio/syr063

[jmor70161-bib-0094] Tudge, C. C. , A. Asakura , and S. T. Ahyong . 2012. “Infraorder Anomura MacLeay, 1838.” In Treatise on Zoology—Anatomy, Taxonomy, Biology. The Crustacea, Volume 9 Part B, edited by F. R. Schram and J. C. von Vaupel Klein , 221–333. Brill.

[jmor70161-bib-0095] Vermeij, G. J. 1975. “Evolution and Distribution of Left‐Handed and Planispiral Coiling in Snails.” Nature 254: 419–420.

[jmor70161-bib-0096] Vermeij, G. J. 2020. “Overcoming the Constraints of Spiral Growth: The Case of Shell Remodelling.” Palaeontology 63: 1035–1047.

[jmor70161-bib-0097] Volker, V. L. 1969. “Zur Morphologie Der Scheren Des Landeinsiedlerkrebses (*Coenobita scaevola* Forskal). Ihre Beziehung Zum Gehäuseverschluß Und Ihre Funktion.” Zoologische Beitrage 15: 203–217.

[jmor70161-bib-0098] Ward, J. H. 1963. “Hierarchical Grouping to Optimize an Objective Function.” Journal of the American Statistical Association 58: 236–244.

[jmor70161-bib-0100] Williams, A. B. 1984. Shrimps, Lobsters, and Crabs of the Atlantic Coast of the Eastern United States. Smithsonian Institution Press.

[jmor70161-bib-0101] Williams, J. D. , and J. J. McDermott . 2004. “Hermit Crab Biocoenoses: A Worldwide Review of the Diversity and Natural History of Hermit Crab Associates.” Journal of Experimental Marine Biology and Ecology 305: 1–128.

[jmor70161-bib-0102] Wolfe, J. M. , J. W. Breinholt , K. A. Crandall , et al. 2019. “A Phylogenomic Framework, Evolutionary Timeline and Genomic Resources for Comparative Studies of Decapod Crustaceans.” Proceedings of the Royal Society B 286: 20190079. 10.1098/rspb.2019.0079. 31014217 PMC6501934

[jmor70161-bib-0103] Wolff, T. 1961. “A Bathyal‐Abyssal Hermit Crab With a Calcified Asymmetrical Abdomen.” Nature 190: 931–932.

[jmor70161-bib-0104] Wright, A. G. , C. L. Griffiths , and T. P. A. Botha . 2020. “Population Structure and Morphology of South African Deep‐Water Parapagurid Hermit Crabs.” Acta Zoologica 101: 227–241.

[jmor70161-bib-0105] Zelditch, M. L. , D. L. Swiderski , H. D. Sheets , and W. L. Fink . 2012. Geometric Morphometrics for Biologists: A Primer. Elsevier Academic Press.

[jmor70161-bib-0106] Zhang, J. , X. Wu , C. He , B. Wu , S. Zhang , and J. Sun . 2024. “Near‐Infrared Spectroscopy Combined With Fuzzy Improved Direct Linear Discriminant Analysis for Nondestructive Discrimination of Chrysanthemum Tea Varieties.” Foods 13: 1439. 10.3390/foods13101439. 38790739 PMC11119828

