# Supplementary material for: On Shell Closure and Beyond: Classification and Functional Interpretation of Chela Types in Paguroidea (Decapoda)
Source: J Morphol. 2026 Aug 18;287(8):e70161. doi: 10.1002/jmor.70161 (PMC13482688; doi:10.1002/jmor.70161)
Supplement: Supplementary file 1 [file JMOR-287-e70161-s002.docx]

**Table S1:** Taxa included in the shape analyses and the side-specific classification of their chelae into different shape types based on Ward’s cluster analysis. Shape types are defined as follows: Type I – compact; type II – sturdy; type III – elongate; type IV – scutiform; type V – discoid; type VI – semidiscoid. F, female; m, male.

| **taxon** | **species** | **catalog #** | **sex** | **symmetry category, handedness** | **shape type**  **(left ; right)** |
| --- | --- | --- | --- | --- | --- |
| Coenobitidae | *Coenobita rugosus*  H. Milne Edwards, 1837 | USNM 1068961 | f | heterochelate, left-handed | - ; II |
|  | *Coenobita scaevola*  (Forskål, 1775) | USNM 97951 | m | heterochelate, left-handed | IV ; II |
| Diogenidae | *Cancellus spongicola*  Benedict, 1901 | USNM 128750 | m | homoiochelate, left-handed | II ; II |
|  | *Clibanarius erythropus*  (Latreille, 1818) | ZSRO YE003 | f | homoiochelate, left-handed | II ; II |
|  | *Diogenes edwardsii*  (De Haan, 1849) | USNM 26289 | m | heterochelate, left-handed | IV ; I |
|  | *Diogenes pugilator*  (Roux, 1829) | ZSRO YE011 | - | heterochelate, left-handed | IV ; I |
|  | *Isocheles pilosus*  (Holmes, 1900) | USNM 49988 | - | homoiochelate, left-handed | V ; V |
|  | *Paguristes digueti*  Bouvier, 1893 | USNM 1071910 | f | homoiochelate, left-handed | IV ; IV |
|  | *Paguristes eremita*  (Linnaeus, 1767) | ZSRO YE029 | m | heterochelate, left-handed | II ; II |
|  | *Paguristes streaensis*  Pastore, 1984 | ZSRO YE025 | f | heterochelate, left-handed | II ; II |
|  | *Paguropsis andersoni*  (Alcock, 1899) | USNM 1442005 | m | homoiochelate, left-handed | I ; I |
| Lithodidae | *Cryptolithodes sitchensis*  Brandt, 1853 | USNM 296455 | m | heterochelate, right-handed | I ; IV |
|  | *Dermaturus mandtii*  Brandt, 1850 | USNM 1267591 | f | heterochelate, right-handed | - ; IV |
|  | *Hapalogaster grebnitzkii*  Schalfeew, 1892 | ZSRO XXXX | - | heterochelate, right-handed | - ; III |
|  | *Hapalogaster mertensii*  Brandt, 1850 | ZSRO E276 | - | heterochelate, right-handed | III ; III |
| Paguridae | *Agaricochirus erosus*  (A. Milne-Edwards, 1880) | USNM 267523 | - | heterochelate, right-handed | I ; V |
|  | *Manucomplanus spinulosus* (Holthuis, 1959) | USNM 275937 | f + m | heterochelate, right-handed | I ; V |
|  | *Pagurus anachoretus*  Risso, 1827 | ZSRO YE017 | - | heterochelate, right-handed | III ; II |
|  | *Pagurus beringanus*  (Benedict, 1892) | ZSRO E275 | - | heterochelate, right-handed | - ; V |
|  | *Pagurus bernhardus*  (Linnaeus, 1758) | ZSRO E121 | f | heterochelate, right-handed | I ; II |
|  | *Pagurus cuanensis*  Bell, 1845 | ZSRO PA 201 | f | heterochelate, right-handed | I ; II |
|  | *Phimochirus holthuisi*  (Provenzano, 1961) | USNM 184297 | f | heterochelate, right-handed | III ; IV |
|  | *Pylopagurus discoidalis*  (A. Milne-Edwards, 1880) | USNM  309727 USNM 309679 | m  m | heterochelate, right-handed | I ; V  I ; I |
| Parapaguridae | *Parapagurus pilosimanus*  Smith, 1879 | USNM 1199725 | f + m | heterochelate, right-handed | III ; IV |
|  | *Probeebei mirabilis*  Boone, 1926 | USNM 267810 | m | homoiochelate, right-handed | III ; III |
|  | *Sympagurus dimorphus*  (Studer, 1883) | ZSRO  SR17 | - | heterochelate, right-handed | III ; IV |
| Pylochelidae | *Bathycheles integer*  (Forest, 1987) | ZSRO  SR348 | - | homochelate | VI ; VI |
|  | *Pomatocheles jeffreysii*  Miers, 1879 | USNM 237343 | - | homochelate | VI ; VI |
|  | *Trizocheles boasi*  Forest, 1987 | ZSRO  SR350 | - | homochelate | - ; II |
| Xylopaguridae | *Xylopagurus cancellarius*  A. Milne-Edwards, 1880 | USNM 1278514 | f | heterochelate, right-handed | III ; IV |

**Table S2:** Overview of the statistical test designs (1 to 6) conducted to investigate how chela shape reflects functional and systematic classifications. Each test is defined by its classification criterion, from which the analyzed groups and the effective sample size (after exclusions) are derived. The corresponding research aim specifies the analytical focus of each test, addressing morphological variation in relation to side-specific dominance, symmetry category (homo-, homoio-, and heterochelate), ecological context, and systematic groupings. L, left; R, right.

| **Test** | **Classification Criterion** | **Grouping (n groups)** | **Sample Size** | **Exclusions / Notes** | **Research Aim / Question** |
| --- | --- | --- | --- | --- | --- |
| **1** | side-specific family-level classification | Diogenidae (L) and (R), Lithodidae (R), Paguridae (L) and (R), Parapaguridae (L) and (R), Pylochelidae **(8 groups)** | 61-7=**54** | Lithodidae (L), Coenobitidae, Xylopaguridae excluded (insufficient sample size); Pylochelidae as one group | Testing for systematic and side-specific functional signal on family-level. |
| **2** | dominance status | dominant, subdominant **(2 groups)** | 61-5=**56** | Pylochelidae excluded | Testing for side-specific functional signal within Paguroidea exhibiting handedness. |
| **3** | chela type | homochelate (semidiscoid), dominant, subdominant **(3 groups)** | 61-1=**60** | *T. boasi* excluded | Testing for the position of homochelate, dominant and subdominant chelae in relation to each other. |
| **4** | chela type | homochelate (semidiscoid), homoiochelate, dominant (heterochelate), subdominant (heterochelate) **(4 groups)** | 61-1=**60** | *T. boasi* excluded; homoiochelate taxa: *C. spongicola, C. erythropus, I. pilosus, P. digueti, P. andersoni, P. mirabilis* | Testing for the position of homochelate, homoiochelate, dominant and subdominant (heterochelate) chelae in relation to each other. |
| **5A** | side-specific dominance status | dominant (L) and (R), subdominant (L) and (R)  **(4 groups)** | 61-5=**56** | Pylochelidae excluded | Testing for systematic signal within Paguroidea exhibiting handedness. |
| **5B** | side-specific dominance status | dominant (L) and (R), subdominant (L) and (R)  **(4** **groups)** | 61-14=**47** | Pylochelidae, Lithodidae and Coenobitidae excluded | Testing for systematic signal within hetero- and homoiochelate taxa (mostly shell dwellers but without shell modification). |
| **5C** | side-specific dominance status | dominant (L) and (R), subdominant (L) and (R)  **(4** **groups)** | 61-20=**41** | Pylochelidae, Lithodidae, Parapaguridae and Coenobitidae excluded | Testing for systematic signal within hetero- and homoiochelate taxa (only shell dwellers without shell modification). |
| **6** | chela type (including side-specific dominance status) | homochelate (semidiscoid), homoiochelate, dominant (heterochelate) (L) and (R), subdominant (heterochelate) (L) and (R) **(6 groups)** | 61-1=**60** | *T. boasi* excluded | Combination of chela type (test 4) and the side-specific dominance status (test 5). |

**Table S3:** Summary of the PCs of the conducted PCA of the extended (A) and original taxon sampling (B). The extended taxon sampling includes seven female and seven male P. bernhardus specimens to test for shape-dependent sexual dimorphism (see Figure S1). Orange cells mark PCs that together account for at least 95% of the total variance.

| **(A) PCs of extended taxon sampling** | | | |  | **(B) PCs of original taxon sampling (61 chelae)** | | | |
| --- | --- | --- | --- | --- | --- | --- | --- | --- |
| **PC** | **eigenvalue** | **variance (%)** | **sum variance (%)** |  | **PC** | **eigenvalue** | **variance (%)** | **sum variance (%)** |
| **1** | 2.8389 | 43.61 | 43.61 |  | **1** | 3.12E-05 | 39.33 | 39.33 |
| **2** | 1.0731 | 16.49 | 60.10 |  | **2** | 1.44E-05 | 18.15 | 57.48 |
| **3** | 0.5494 | 8.44 | 68.54 |  | **3** | 7.28E-06 | 9.16 | 66.64 |
| **4** | 0.3893 | 5.98 | 74.52 |  | **4** | 5.49E-06 | 6.91 | 73.55 |
| **5** | 0.2880 | 4.43 | 78.95 |  | **5** | 4.27E-06 | 5.38 | 78.93 |
| **6** | 0.2602 | 4.00 | 82.95 |  | **6** | 3.22E-06 | 4.05 | 82.98 |
| **7** | 0.2220 | 3.41 | 86.36 |  | **7** | 2.26E-06 | 2.84 | 85.82 |
| **8** | 0.1970 | 3.03 | 89.38 |  | **8** | 1.74E-06 | 2.19 | 88.01 |
| **9** | 0.1365 | 2.10 | 91.48 |  | **9** | 1.11E-06 | 1.39 | 89.41 |
| **10** | 0.0952 | 1.46 | 92.94 |  | **10** | 1.06E-06 | 1.33 | 90.74 |
| **11** | 0.0879 | 1.35 | 94.29 |  | **11** | 7.68E-07 | 0.97 | 91.71 |
| **12** | 0.0681 | 1.05 | **95.34** |  | **12** | 7.57E-07 | 0.95 | 92.66 |
| 13 | 0.0066 | 1.01 | 96.35 |  | **13** | 7.37E-07 | 0.93 | 93.59 |
| 14 | 0.0344 | 0.53 | 96.88 |  | **14** | 6.00E-07 | 0.76 | 94.34 |
| 15 | 0.0316 | 0.49 | 97.37 |  | **15** | 4.29E-07 | 0.54 | 94.88 |
| 16 | 0.0273 | 0.42 | 97.79 |  | **16** | 4.23E-07 | 0.53 | **95.42** |
| 17 | 0.0248 | 0.38 | 98.17 |  | 17 | 4.15E-07 | 0.52 | 95.94 |
| 18 | 0.0209 | 0.32 | 98.49 |  | 18 | 3.80E-07 | 0.48 | 96.42 |
| 19 | 0.0019 | 0.29 | 98.78 |  | 19 | 3.11E-07 | 0.39 | 96.81 |
| 20 | 0.0167 | 0.26 | 99.03 |  | 20 | 2.91E-07 | 0.37 | 97.18 |
| 21 | 0.0013 | 0.19 | 99.23 |  | 21 | 2.47E-07 | 0.31 | 97.49 |
| 22 | 0.0125 | 0.19 | 99.42 |  | 22 | 2.46E-07 | 0.31 | 97.8 |
| 23 | 0.0114 | 0.18 | 99.59 |  | 23 | 2.44E-07 | 0.31 | 98.1 |
| 24 | 0.0092 | 0.14 | 99.73 |  | 24 | 2.30E-07 | 0.29 | 98.39 |
| 25 | 0.0077 | 0.12 | 99.85 |  | 25 | 2.10E-07 | 0.26 | 98.66 |
| 26 | 0.0033 | 0.05 | 99.91 |  | 26 | 1.81E-07 | 0.23 | 98.89 |
| 27 | 0.0022 | 0.03 | 99.94 |  | 27 | 1.62E-07 | 0.20 | 99.09 |
| 28 | 0.0021 | 0.03 | 99.97 |  | 28 | 1.47E-07 | 0.18 | 99.27 |
| 29 | 0.0012 | 0.02 | 99.99 |  | 29 | 1.26E-07 | 0.16 | 99.43 |
| 30 | 0.0007 | 0.01 | 100 |  | 30 | 1.17E-07 | 0.15 | 99.58 |
|  |  |  |  |  | 31 | 9.61E-08 | 0.12 | 99.7 |
|  |  |  |  |  | 32 | 7.16E-08 | 0.09 | 99.79 |
|  |  |  |  |  | 33 | 6.54E-08 | 0.08 | 99.87 |
|  |  |  |  |  | 34 | 5.36E-08 | 0.07 | 99.94 |
|  |  |  |  |  | 35 | 4.56E-08 | 0.06 | 100 |

**Table S4:** Results of the MANOVA (A) and one-way PERMANOVA (B) of the side-specific classification at family level (**test 1**). *F*-values are colorized green, *p*-values orange, and squared Mahalanobis distances (*MD²*) blue. Overall *F*-values can be found in the top-left cell. *Fail* entries refer to insufficient sample size, while *NAN* (not a number) refers to mathematical artifacts resulting from non-normally distributed data groups. Significant differences between groups are marked with bold *p*-values. Pag, Paguridae; Pylo, Pylochelidae; Dio, Diogenidae; Litho, Lithodidae; Para, Parapaguridae; L, left; R, right.

| **A** *F*: 9.03 | Pag (L) | Pag (R) | Pylo | Dio (L) | Dio (R) | Litho (R) | Para (L) | Para (R) |
| --- | --- | --- | --- | --- | --- | --- | --- | --- |
| Pag (L) | *p*-value | 0.386 | *fail* | 0.52 | 0.458 | *fail* | *fail* | *fail* |
| Pag (R) | 23.521 |  | *fail* | 0.185 | 0.214 | *fail* | *fail* | *fail* |
| Pylo | *fail* | *fail* |  | *fail* | *fail* | *fail* | *fail* | *fail* |
| Dio (L) | 26.98 | 29.719 | *fail* |  | 6.303 | *fail* | *fail* | *fail* |
| Dio (R) | 28.256 | 28.383 | *fail* | 9.121 |  | *fail* | *fail* | *fail* |
| Litho (R) | *fail* | *fail* | *fail* | *fail* | *fail* |  | *fail* | *fail* |
| Para (L) | *fail* | *fail* | *fail* | *fail* | *fail* | *fail* |  | *fail* |
| Para (R) | *fail* | *fail* | *fail* | *fail* | *fail* | *fail* | *fail* | *MD²* |

| **B** *F*: 2.4 | Pag (L) | Pag (R) | Pylo | Dio (L) | Dio (R) | Litho (R) | Para (L) | Para (R) |
| --- | --- | --- | --- | --- | --- | --- | --- | --- |
| Pag (L) | *p*-value | 1 | **0.003** | 1 | 1 | **0.003** | **0.003** | **0.003** |
| Pag (R) | 1.127 |  | 1 | 1 | 1 | **0.003** | 1 | **0.003** |
| Pylo | *NAN* | 0.825 |  | 1 | 1 | **0.003** | **0.003** | **0.003** |
| Dio (L) | 1.061 | 1.128 | 0.711 |  | 1 | **0.003** | **0.003** | **0.003** |
| Dio (R) | 1.041 | 1.025 | 0.914 | 1.053 |  | **0.003** | **0.003** | 1 |
| Litho (R) | *NAN* | *NAN* | *NAN* | *NAN* | *NAN* |  | **0.003** | **0.003** |
| Para (L) | *NAN* | 0.472 | *NAN* | *NAN* | *NAN* | *NAN* |  | **0.003** |
| Para (R) | *NAN* | *NAN* | *NAN* | *NAN* | 1.878 | *NAN* | *NAN* | *F*-value |

**Table S5:** Euclidean distances between group centroids of dominant, subdominant, homochelate, and homoiochelate chelae based on LDA (**test 4**). The longest distance is marked in bold.

|  | subdominant | dominant | homochelate | homoiochelate |
| --- | --- | --- | --- | --- |
| subdominant | 0 | 4.06 | 5.49 | 3.4 |
| dominant | 4.06 | 0 | 6.28 | 2.65 |
| homochelate | 5.49 | 6.28 | 0 | **6.53** |
| homoiochelate | 3.4 | 2.65 | **6.53** | 0 |

**Table S6:** Euclidean distances between group centroids of heterochelate dominant left and right, heterochelate subdominant left and right, homochelate and homoiochelate chelae based on LDA (**test 6**). L, left; R, right.

|  | subdom (L) | dominant (R) | homochelate | homoiochelate | subdom (R) | dominant (L) |
| --- | --- | --- | --- | --- | --- | --- |
| subdom (L) | 0 | 4.92 | 5.52 | 3.86 | 6.56 | 4.83 |
| dominant (R) | 4.92 | 0 | 6.24 | 3.39 | 7.39 | 5.62 |
| homochelate | 5.52 | 6.24 | 0 | 7.05 | 6.31 | 6.37 |
| homoiochelate | 3.86 | 3.39 | 7.05 | 0 | 5.47 | 4.78 |
| subdom (R) | 6.56 | 7.39 | 6.31 | 5.47 | 0 | 4.50 |
| dominant (L) | 4.83 | 5.62 | 6.37 | 4.78 | 4.50 | 0 |

**Figure S1:** Ward’s cluster analysis of the extended taxon sampling containing 7 additional female (blue) and male (red) specimens of *P. bernhardus.* The analysis is based on the first 12 PCs that account for over 95% of variance of the PCA of the extended taxon sampling (see Table S3A). No sex-specific shape differences for the dominant and subdominant chelae in *P. bernhardus* are depicted. Furthermore, MANOVA and PERMANOVA yield no significant separation between female and male chelae (*p* = 1), approving the results of the cluster analysis.
